# Identification of Biomarkers of Human Skin Ageing in Both Genders. Wnt Signalling - A Label of Skin Ageing?

**DOI:** 10.1371/journal.pone.0050393

**Published:** 2012-11-30

**Authors:** Evgenia Makrantonaki, Thore C. Brink, Vasiliki Zampeli, Rana Mohsen Elewa, Barbara Mlody, Amir M. Hossini, Bjoern Hermes, Ulf Krause, Juergen Knolle, Marwa Abdallah, James Adjaye, Christos C. Zouboulis

**Affiliations:** 1 Departments of Dermatology, Venereology, Allergology and Immunology, Dessau Medical Center, Dessau, Germany; 2 Institute of Clinical Pharmacology and Toxicology, Charité Universitaetsmedizin Berlin, Berlin, Germany; 3 Department of Vertebrate Genomics, Max Planck Institute for Molecular Genetics, Berlin, Germany; 4 Department of Dermatology, Andrology and Venereology, Faculty of Medicine, Ain-Shams University, Hassan Ibrahim Hassan, Nasr City, Cairo, Egypt; 5 Department of Pathology, Dessau Medical Center, Dessau, Germany; 6 The Stem Cell Unit, Department of Anatomy, College of Medicine, King Saud University, Riyadh, Saudi Arabia; The Centre for Research and Technology, Greece

## Abstract

The goal of our work has been to investigate the mechanisms of gender-independent human skin ageing and examine the hypothesis of skin being an adequate model of global ageing. For this purpose, whole genome gene profiling was employed in sun-protected skin obtained from European Caucasian young and elderly females (mean age 26.7±4 years [n1 = 7] and 70.75±3.3 years [n2 = 4], respectively) and males (mean age 25.8±5.2 years [n3 = 6] and 76±3.8 years [n4 = 7], respectively) using the Illumina array platform. Confirmation of gene regulation was performed by real-time RT-PCR and immunohistochemistry. 523 genes were significantly regulated in female skin and 401 genes in male skin for the chosen criteria. Of these, 183 genes exhibited increased and 340 decreased expression in females whereas 210 genes showed increased and 191 decreased expression in males with age. In total, 39 genes were common in the target lists of significant regulated genes in males and females. 35 of these genes showed increased (16) or decreased (19) expression independent of gender. Only 4 overlapping genes (*OR52N2*, *F6FR1OP2*, *TUBAL3* and *STK40*) showed differential regulation with age. Interestingly, Wnt signalling pathway showed to be significantly downregulated in aged skin with decreased gene and protein expression for males and females, accordingly. In addition, several genes involved in central nervous system (CNS) ageing (f.i. *APP*, *TAU*) showed to be expressed in human skin and were significanlty regulated with age. In conclusion, our study provides biomarkers of endogenous human skin ageing in both genders and highlight the role of Wnt signalling in this process. Furthermore, our data give evidence that skin could be used as a good alternative to understand ageing of different tissues such as CNS.

## Introduction

Ageing is a complex process and underlies multiple influences with the probable involvement of heritable and various environmental factors. Several theories have been conducted regarding the pathomechanisms of ageing including cellular senescence and decreased proliferative ability [1], reduction of cellular DNA repair capacity, loss of telomeres with advancing age [2], point mutations of extranuclear mtDNA [3] which may be associated to increased oxidative stress [4] and increased frequency of chromosomal abnormalities [5]. Complementary, the importance of hormones and their metabolism on the ageing process has been proposed by recent studies performed on animal and in vitro models [6], [7], [8].

Despite these advances our knowledge on human ageing still remains limited. This may be causally related to the fact that the collection of human specimens from internal organs throughout life for experimental research purposes is associated with major practical and ethical obstacles. Alternatively, the use of skin as a common research tool may offer a promising approach. With age skin undergoes major morphological and physiological changes [9]. In sun-protected nonexposed skin areas ageing is mainly attributed to intrinsic factors such as genetics and changes in the endocrine environment and reflects degradation processes of the entire organism [10], [11]. In particular, recent data obtained from human epithelial skin cells indicated the use of skin as a model of investigating ageing of tissues derived of the same embryologic origin such as central nervous system (CNS) [10].

In current years, microarray technology has become a valuable tool for screening genetic material of several model organisms ranging from yeast and nematodes to mice in order to map genes and pathways which are involved in the pathogenesis of ageing and extension of lifespan. In the mouse, a database, that catalogues changes in gene expression as a function of age has been recently established [12]. Studies to investigate changes in gene expression during human ageing have been performed on naturally aged human foreskin obtained from children and elderly males. Some of the mechanisms proposed to be involved in the induction of skin ageing comprise disturbed lipid metabolism, altered insulin and STAT3 signalling, upregulation of apoptotic genes partly due to the deregulation of FOXO1, downregulation of members of the jun and fos family, differential expression of cytoskeletal proteins (e.g. keratin 2A, 6A, and 16A), extracellular matrix components (e.g. PI3, S100A2, A7, A9, SPRR2B), and proteins involved in cell-cycle control (e.g. CDKs, GOS2) [13]. Additionally, recent data obtained by studies of progeroid syndromes (e.g. Hutchinson-Gilford progeria, Werner syndrome, Rothmund-Thomson syndrome, Cockayne syndrome, ataxia teleangiectasia, and Down syndrome) illustrate that among the most important biological processes involved in skin ageing are alterations in DNA repair and stability, mitochondrial function, cell cycle and apoptosis, ubiquitin-induced proteolysis, and cellular metabolism reviewed in Capell et al [14]. Array technology has been also used for the analysis of complex DNA methylation patterns in skin samples obtained from healthy and elderly donors showing a statistically significant trend towards DNA hypermethylation in aged samples [15].

However, no study has so far documented the gender-independent differences in the expression profiling of aged skin between young and elderly European Caucasian adults. Therefore, we investigated the profile of genes expressed in sun-protected skin of young and elderly females (mean age 26.7±4 years [n1 = 7] and 70.75±3.3 years [n2 = 4], respectively) and males (mean age 25.8±5.2 years [n3 = 6] and 76±3.8 years [n4 = 7], respectively) when circulating hormones reach their maximum levels in blood and after the onset of hormone decline, accordingly. In addition, the expression of genes involved in the pathogenesis of neurodegenerative diseases such as Morbus Alzheimer and Morbus Parkinson have been further investigated in order to examine the hypothesis that skin is an adequate model of CNS ageing.

## Results

### Morphology of skin biopsies in young and old male and female donors

**Figure 1:**
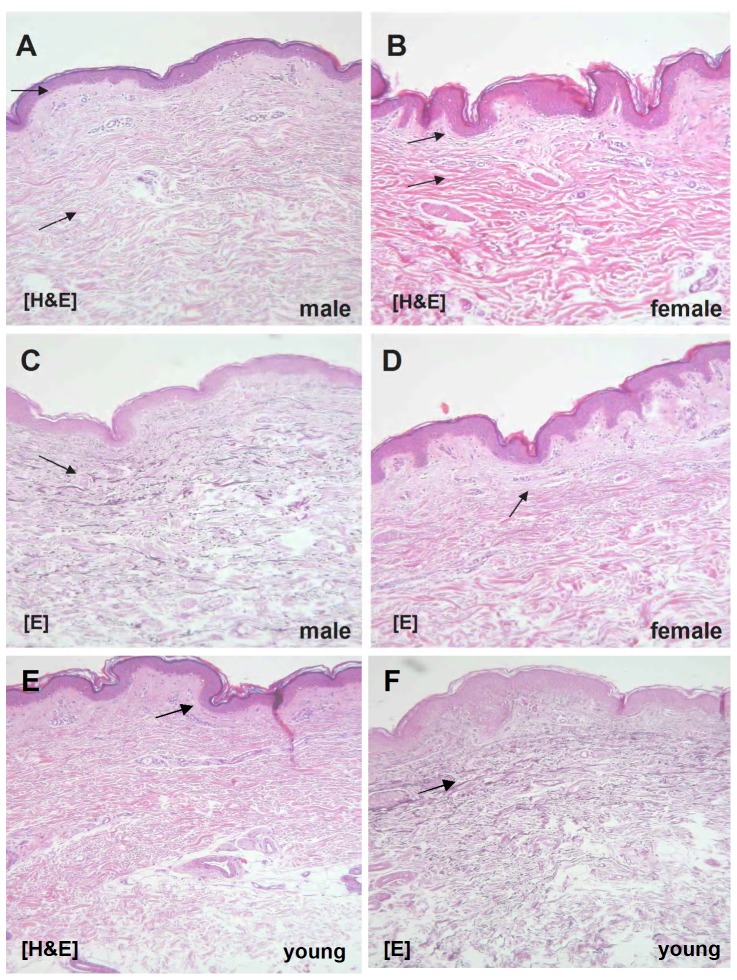
A comparison between male (A, C) and female (B, D) endogenous aged skin. Staining via hematoxylin eosin (A, B) and elastica staining (C, D), respectively, revealed that the dermis in the male is significantly thicker than in the female. In contrast, epidermis and subcutaneous tissue are significantly thicker in the female. Skin derived by a young donor after hematoxylin (E) and elastica (F) staining.

A comparison between male and female sun-protected skin derived from the inner side of the upper arm revealed that the male dermis is much thicker than the female one (1.8-fold, p<0.05). In contrast, epidermis and subcutaneous tissue is thicker in the female (3.5-fold, p<0.05 and 10-fold, respectively) [Figure 1].

### Global gene expression profiling of skin ageing via cDNA microarrays

We provide experimental data at the molecular level based on a genomics approach to investigate changes in gene expression during human skin ageing. To identify and further analyse common and gender-specific genes, pathways and biological processes altered with age in human skin, RNA was isolated from skin biopsies of young and old male and female healthy volunteers. Whole-genome gene expression analysis employing the Illumina Bead Chip technology was then used to profile the transcriptome of these biopsies. We used the rank invariant method for normalisation and the t-test error model.

Data reproducibility is demonstrated by sample correlation and clustering (Figure S1). As expected the clustering of the samples shows a good correlation for most of the replicates (>0.9). The correlation coefficients for replicates of the 6 young and 7 old males and the 7 young females show in most cases high correlations. Additionally, the transcriptional difference between young and old males is minor and even the correlation of all males to the young females is high. However, f.i. 3 of the 4 old females exhibit a little lower correlation to each other as well as to young females and all males. Old female number 1 shows the worse correlation to the rest (ranging from 0.67–0.87). These differences could either reflect biological heterogeneity between samples or technical variations during the whole process of sample collection, RNA isolation and hybridisation.

**Figure 2:**
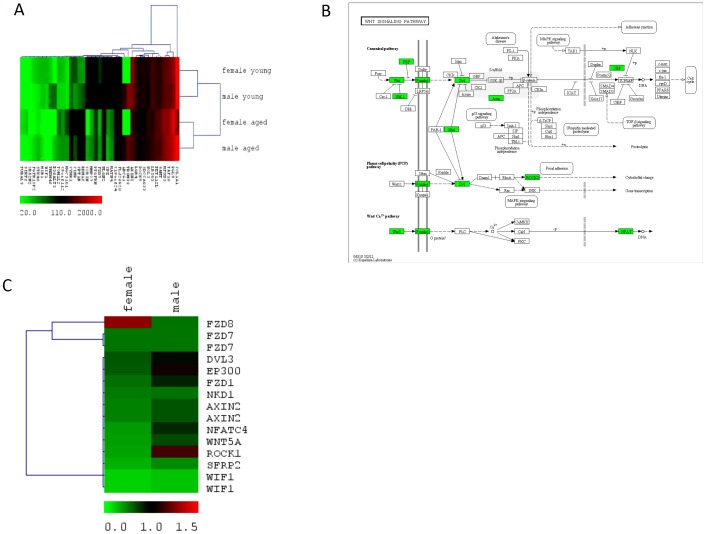
Hierarchical clustering of genes that overlap in aged samples, irrespective of sex (A); Age dependent changes in WNT signalling of males and females (B); Hierachical clustering showing a similar expression profile in young subjects for genes regulated by age in WNT-signalling (C). (A) All relevant genes are listed according to their average expression values. Each column corresponds to one gene, each row to 4 replicates for each group sample. Green indicates a low expression value and red a high expression value. Black corresponds to a median expression value in relation to all shown values. Euclidean Distance was used to calculate average linkage clustering of the sample and gene tree. (B) The KEGG-pathway shows genes which are negative regulated with age in green colour. (C) All relevant genes are listed according to their expression values shown as log2. Each row corresponds to one gene, each column to 4 different microarray experiments. Green indicates a low expression value and red a high expression values. Black corresponds to a median expression value in relation to all shown values.

Normalised data were analysed for significant (detection >0.99 for at least one group and p-value<0.05) changes in gene expression between young and old females and males with ratios of 1.3 and above (full data provided in Table S1 and S2, accordingly). For the chosen criteria, we found more regulated genes in females (523) than in males (401). Of these, 183 genes exhibited increased and 340 decreased expression in females, whereas 210 genes showed increased and 191 decreased expression in males with age. For further analysis, these two target gene lists were divided in genes with increased and decreased expression with age. The total numbers are shown in Figure S1. Figure 2A shows the hierarchical clustering of top 50 regulated genes showing a sex independent expression profile in young subjects for genes regulated with age.

### Functional annotation of expressed genes

The age-related gene lists (corresponding Gene accession as input) were analysed using the Gene Annotation Tools – DAVID (http://niaid.abcc.ncifcrf.gov/) and FatiGO+ (http://babelomics.bioinfo.cipf.es/fatigoplus/cgibin/fatigoplus.cgi) to identify altered pathways and Gene Ontologies (GOs). The complete list of significant age-regulated GOs (Biological Process, Cellular Compartment, Molecular Function) in both genders, respectively, are presented in Tables S3 and S4.

Up-regulated expression in female skin biopsies correlated with increases for example in translation (11), tRNA aminoacylation (4) and RNA processing (11), with increased expression of genes related to cellular compartments f.i. mitochondrion (24), microtubule associated complex (5), cytosol (21) and ribosome (6) and to molecular functions f.i. cytoskeletal protein binding (12), ATP binding (23), ribonucleotide binding (27) and nucleotide binding (31) (Table S3). Significantly down-regulated biological processes in female skin were f.i. translational elongation (34), extracellular matrix (19) and structure organisation (21), cell adhesion (31), ribosome biogenesis (10), neuron differentiation (17), neuron projection development (11), protein ubuiqitination (7), regulation of cell proliferation (24), positive regulation of immune system process (10) and regulation of transcription (63). Furthermore, downregulated expression of genes involved in cellular compartments such as cytosolic ribosome (31), ribosomal subunit (31), extracellular matrix (31) and extracellular region (67) was observed. Molecular functions f.i. structural constituent of ribosome (31), structural molecule activity (43), RNA binding (31) and transcription factor activity (34) were also downregulated.

In male skin biopsies we observed an increase in the number of genes involved in biological processes such as protein (14) and lipid (6) catabolic process, macromolecule catabolic process (16), organophosphate metabolic process (7) and vitamin transport (3). Genes influencing the cellular compartments were upregulated such as cell (22) and membrane (16) fraction, nuclear telomeric heterochromatin (2), synapse part (7), synaptic vesicle (4) and soluble fraction (8). Molecular functions such as protein/serine threonine kinase activity (10), oxidoreductase activity (4) and identical protein binding (13) were upregulated. On the other hand, biological processes f.i. regulation of transcription (39), melanin metabolic process (3) and pigmentation (5), regulation of transcription, DNA-dependent (28) and regulation of RNA metabolic process (28) were downregulated. Genes involved in cellular compartments such as extracellular region part (15) and external side of plasma membrane (6) showed a downregulation. Finally, molecular functions including zinc ion binding (44), transition metal ion binding (48), cation (62), ion (62), GTP (9) and purine nucleotide binding (29) were downregulated.

### Signalling and metabolic pathways

For down-regulated genes in female skin, Kegg pathways such as ribosome (32), cell adhesion molecules (CAMS) (9), ECM-receptor interaction (8), focal adhesion (12) melanogenesis (8), basal cell carcinoma (6) and WNT signalling pathway (10) were found to be significantly regulated at the <0.1 level (Table 1). In male skin biopsies significantly up-regulated Kegg pathways were f.e. the arginine and proline metabolism (4), insulin signalling (5) and acute myeloid leucemia (5), whereas downregulated were melanogenesis (6), gap junction (4), ubiquitin-mediated proteolysis (4), basal cell carcinoma (3) and WNT signalling pathway (5) (Table 1). The full lists of pathways of all regulated genes (up-and downregulated) in both genders are shown in Tables S5 and S6, accordingly.

**Table 1:** Results of the analysis of statistically significant pathways at *P*-value<0.1 in skin biopsies of males and females with age.

	Female		Male	
Pathway Description	down	up	down	up
hsa05221:Acute myeloid leukemia	-	-	-	5
hsa00330:Arginine and proline metabolism	-	-	-	4
hsa04910:Insulin signalling pathway	-	-	-	5
hsa04916:Melanogenesis	8	-	6	-
hsa04310:Wnt signalling pathway	10	-	5	-
hsa04514:Gap junction	-	-	4	-
hsa05217:Basal cell carcinoma	6	-	3	-
hsa05130:Pathogenic Escherichia coli infection	-	-	3	-
hsa04120:Ubiquitin mediated proteolysis	-	-	4	-
hsa03010:Ribosome	32	-	-	-
hsa04512:ECM-receptor interaction	8	-	-	-
hsa04510:Focal adhesion	12	-	-	-
hsa04514:Cell adhesion molecules (CAMs)	9	-	-	-
hsa05210:Colorectal cancer	6	-	-	-
hsa04640:Hematopoietic cell lineage	6	-	-	-
hsa04670:Leukocyte transendothelial migration	7	-	-	-

Pathways were taken from the KEGG database (human pathways). Genes that were judged as non-detectable by the BG value criterion were excluded from analysis (up: number of genes that were taken into account for computing the statistical test showing upregulated expression with age; down: number of genes that were taken into account for computing the statistical test showing downregulated expression with age in females and males, respectively). The full lists of pathways of all regulated genes (up-and downregulated) in both genders are shown in Table S5 and S6, accordingly.

### Common patterns of ageing in male and female skin tissues

The only significant regulated overlapping pathways in both genders were the WNT signalling pathway (Figure 2B, 2C), the basal cell carcinoma pathway and the melanogenesis pathway. In total, 39 genes were common in the target lists of significant regulated genes in males and females (Figure S1, Table 2). 35 of these genes showed increased (16) or decreased (19) expression independent of gender. Only 4 genes showed different regulation in both genders. *OR52N2, FGFR1OP2 and STK40* showed increased expression in male and decreased expression in female aged skin, whereas *TUBAL3* decreased expression in male and increased expression in females with age.

**Table 2:** Thirty-nine age-related genes which are significantly up- (16) or downregulated (19) with age in our data in both genders (*P*-value≤0.05; detection ratio 1.3).

Name	Accession Nr.	Description	Ratio/male	*P* -value	Ratio/female	*P*-value
*SIRT6*	NM_016539.1	sirtuin (silent mating type information regulation 2 homolog) 6 S.cerevisiae	**1.246**	0.030	**1.762**	0.027
*RDH16*	NM_003708.2	Homo sapiens retinol dehydrogenase 16 (all-trans and 13-cis) (RDH16), mRNA.	**1.016**	0.014	**1.186**	0.048
*CPT1B*	NM_152246.1	carnitine palmitoyltransferase 1B (muscle), transcript variant 3	**1.000**	0.013	**1.021**	0.007
*MGC3101*	NM_024043.2	Homo sapiens hypothetical protein MGC3101 (MGC3101), mRNA.	**0.796**	0.001	**1.446**	0.043
*C9orf112*	NM_138778.1	Homo sapiens chromosome 9 open reading frame 112 (C9orf112), mRNA.	**0.690**	0.000	**0.479**	0.050
*STK40*	NM_032017.1	Homo sapiens serine/threonine kinase 40 (STK40), mRNA.	**0.621**	0.011	−0.419	0.023
*TOM1L2*	NM_001033551.1	Homo sapiens target of myb1-like 2 (chicken) (TOM1L2), transcript variant 1, mRNA.	**0.601**	0.001	**0.674**	0.033
*CINP*	NM_032630.2	cyclin-dependent kinase2-interacting protein	**0.584**	0.002	**0.503**	0.029
*PGLS*	NM_012088.2	Phosphogluconolactonase	**0.541**	0.000	**0.533**	0.028
*FLJ20920*	NM_025149.3	hypothetical protein FLJ20920	**0.519**	0.009	**0.604**	0.009
*CYHR1*	NM_032687.2	cysteine/histidine-rich 1	**0.511**	0.016	**0.708**	0.002
*TAF10*	NM_006284.2	TAF10RNA polymerase II, TATA box binding protein (TBP)-associated factor, 30 kDa	**0.503**	0.024	**0.385**	0.014
*GAMT*	NM_000156.4	Homo sapiens guanidinoacetate N-methyltransferase (GAMT), transcript variant 1, mRNA.	**0.496**	0.003	**0.738**	0.042
*NOL3*	NM_003946.3	nucleolar protein 3 (apoptosis repressor with CARD domain)	**0.487**	0.016	**0.476**	0.050
*PET112L*	NM_004564.1	PET112-like (yeast)	**0.472**	0.005	**0.617**	0.010
*OR52N2*	NM_001005174.1	Homo sapiens olfactory receptor, family 52, subfamily N, member 2 (OR52N2), mRNA.	**0.458**	0.001	−0.403	0.018
*MFSD3*	NM_138431.1	Homo sapiens major facilitator superfamily domain containing 3 (MFSD3), mRNA.	**0.452**	0.028	**0.595**	0.034
*C19orf24*	NM_017914.2	Homo sapiens chromosome 19 open reading frame 24 (C19orf24), mRNA.	**0.395**	0.049	**0.627**	0.043
*FGFR1OP2*	NM_015633.1	Homo sapiens FGFR1 oncogene partner 2 (FGFR1OP2), mRNA.	**0.384**	0.013	−0.590	0.001
*TRIM33*	NM_033020.2	Homo sapiens tripartite motif-containing 33 (TRIM33), transcript variant b, mRNA.	−0.396	0.002	−0.515	0.048
*SDCCAG33*	NM_005786.3	serologically defined colon cancer antigen 33	−0.397	0.017	−0.699	0.003
*LRIG3*	NM_153377.3	Homo sapiens leucine-rich repeats and immunoglobulin-like domains 3 (LRIG3), mRNA.	−0.398	0.002	−0.650	0.029
*DOCK9*	NM_015296.1	Homo sapiens dedicator of cytokinesis 9 (DOCK9), mRNA.	−0.415	0.014	−0.815	0.009
*ABCG1*	NM_004915.3	ATP-binding cassette, subfamily G (WHITE),member 1, transcript variant 4	−0.434	0.028	−0.577	0.046
*NLGN2*	NM_020795.2	Homo sapiens neuroligin 2 (NLGN2), mRNA.	−0.436	0.025	−0.476	0.049
*LGR4*	NM_018490.1	leucine-rich repeat-containing G protein-coupled receptor 4	−0.442	0.000	−0.417	0.028
*PTGFRN*	NM_020440.2	Homo sapiens prostaglandin F2 receptor negative regulator (PTGFRN), mRNA.	−0.457	0.003	−0.681	0.035
*MIB1*	NM_020774.2	mindbomb homolog 1 (Drosophila)	−0.539	0.002	−0.935	0.019
*B3GALT3*	NM_003781.2	Homo sapiens UDP-Gal:betaGlcNAc beta 1,3-galactosyltransferase, polypeptide 3 (B3GALT3), transcript variant 1, mRNA.	−0.590	0.043	−0.575	0.043
*AXIN2*	NM_004655.2	axin 2 (conductin, axil)	−0.617	0.008	−1.039	0.006
*FZD7*	NM_003507.1	Homo sapiens frizzled homolog 7 (Drosophila)	−0.915	0.045	−0.910	0.026
*TUBAL3*	NM_024803.1	Homo sapiens tubulin, alpha-like 3 (TUBAL3), mRNA.	−0.915	0.029	**0.673**	0.043
*MMP27*	NM_022122.2	matrix metalloprotease 27	−0.982	0.048	−1.579	0.001
*COL1A1*	NM_000088.2	collagen type 1, alpha 1	−1.144	0.038	−2.349	0.001
*MATN4*	NM_003833.2	matrilin 4, transcript variant 1	−1.393	0.014	−1.823	0.003
*TMEM46*	NM_001007538.1	transmembrane protein 46	−1.436	0.026	−1.626	0.038
*CPZ*	NM_001014447.1	carboxypeptidase Z, transcript variant 1	−1.674	0.008	−1.945	0.014
*WIF1*	NM_007191.2	WNT inhibitory factor 1	−1.929	0.026	−2.247	0.019
*CORIN*	NM_006587.2	Homo sapiens corin, serine peptidase (CORIN), mRNA.	−1.935	0.035	−3.216	0.014

The logarithmic ratios with base two (log_2_) and corresponding *P*-values, accordingly, are listed. Only 4 genes showed different regulation in both genders. The full lists with all regulated genes in females and males, respectively are in Tables S1 and S2.

### Confirmation of microarray data via real time RT-PCR

**Figure 3:**
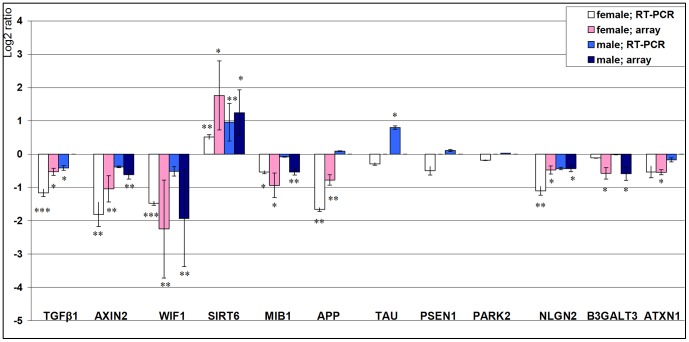
Confirmation of microarray data via real time RT-PCR. Examination of expression levels of candidate genes: *TGFβ*, *AXIN2*, *WIF1*, *SIRT6*, *MIB1*, *APP*, *TAU*, *PSEN1*, *PARK2*, *NLGN2*, *B3GALT3* and *ATXN1* in female and male young and elderly donors. The figure shows the logarithmic ratios aged vs. young with base two (log2) of the selected genes whose expression was deduced by microarray and real-time RT–PCR. A ratio of 1 represents a twofold change with age. Values greater than zero mean higher expression in aged and values less than zero, higher expression in young donors. All experiments have been performed in triplicate. (p<0.05: *, p<0.01:**, p<0.001: ***).

The genes selected to be further investigated in the current study, were selected according to their proved role in ageing in other tissues or species, their effect on proliferation and differentiation of skin cells as well as influence on tumour physiology, chronic inflammation and age related diseases such as atherosclerosis, Alzheimer's and Parkinson's disease [9], [16], [17], [18], [19], [20]. Examination of expression levels of *TGFβ*, *AXIN2*, *WIF1*, *SIRT6*, *MIB1*, *B3GALT3*, *APP*, *TAU*, *PSEN1*, *PARK2*, *ATXN1*, and *NLGN2* (Figure 3) was performed. messenger RNA gene expression of the young female and male donors, respectively, was set as control at 100%, and mRNA gene expression in elderly donors was calculated as the percentage of the change from control. Expression of *TGFβ* was significantly downregulated in female and male aged skin (45%; p<0.001 and 75%; p<0.05, accordingly). *WIF1* expression was significantly downregulated in female and male aged skin (36%; p<0.01 and 69%; p<0.01, respectively), whereas *SIRT6* expression was significanlty upregulated in aged skin in both sexes (143%; p<0.05 and 194%; p<0.01, respectively), correlating to the array data. *MIB1*, *AXIN2*, *NLGN2* and *APP* were significantly downregulated only in female aged skin (69%; p<0.05, 29%; p<0.01, 47%; p<0.01; 32%, p<0.01, respectively). *TAU* was significantly upregulated only in male aged skin (177%; p<0.05). *B3GALT3*, *PSEN1*, *PARK2*, and *ATXN1* showed to be expressed in human skin, however no significant changes were observed with age (Figure 3).

### Expression of genes associated with ageing at protein level via immunohistochemistry

**Figure 4:**
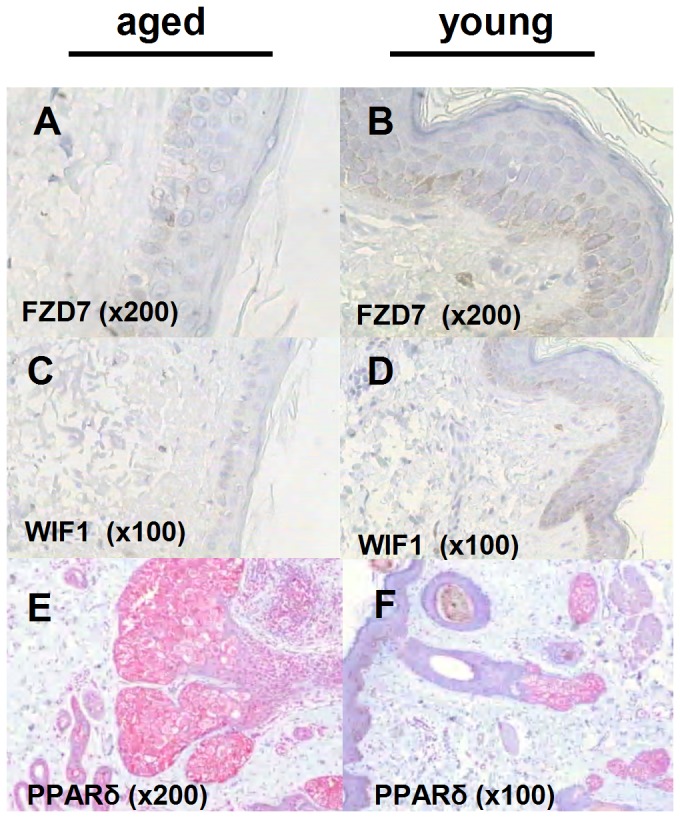
Protein expression of target genes via immunohistochemistry. A comparison between young and aged sun-protected skin provided by female and male healthy donors (n = 7, accordingly). Localization of WIF1 (A,B) and FZD7 (C,D) in aged and young skin, respectively [DAB staining, Dako]. The expression of both proteins was negative in skin biopsies obtained from elderly subjects. On the other hand, the young group showed a significant higher expression of both proteins (p = 0.0019 and p = 0.013, respectively) only in the basal cell layer of the epidermis. No significant gender differences were observed. E, F: Localization of PPAR-δ in aged and young skin, respectively [LSAB, REAL Detection System, Dako]. Strong immune reaction of the sebaceous glands in the skin of both groups and significantly stronger reaction of the sebaceous duct in the skin of the aged group (p<0.019). All experiments have been performed in triplicate.

Following antigens were examined at protein level: FZD7, WIF-1 and PPAR-δ. The tested antigens were expressed in almost all skin structures but showed a differential expression according to age (Figure 4). The expression of FZD7 and WIF1 was negative in skin biopsies obtained from elderly subjects. On the other hand, the young group showed a significant higher expression of both proteins (p = 0.0019 and p = 0.013, respectively) only in the basal cell layer of the epidermis. No significant gender differences were observed (Figure 4A, B, C, D). Sebaceous glands showed the highest PPARδ expression amongst other skin structures, followed by sweat glands (Figure 4E, F). The staining was differentiation-dependent. Epidermis also showed positive PPARδ expression in the form of focal or homogenous weak staining. PPARδ expression was in all cases confined to superficial and mid epidermal layers. The intensity of staining in sebaceous ducts was positively correlated to age (p<0.019).

## Discussion

Androgens may play a substantial role in skin morphology. This fact has been described in several animal and human studies, which have documented gender-specific characteristics of the skin structure [21], [22]. Our findings correspond to previous studies showing that in humans, dermis in intrinsically aged male skin is significantly thicker than in female aged skin, while females have thicker subcutaneous tissue [22]. These data provide evidence that androgens and their decline with age may account for the regulation of dermis.

The results of the analyses in human skin biopsies in males and females revealed a higher degree of regulation in females compared to males, especially for genes down-regulated with age. Although there is no definitive explanation for this difference, the influence of varying hormone levels in males and females, and additionally the sex-specific decline with age, might account for the observed variations. In this regard, it should be kept in mind that the female volunteers did not exhibit synchronised period upon sample collection but that the group of old females were selected to be post-menopausal. In our data, analyses on the single gene level revealed only 39 overlapping genes between male (401) and female (523) skin ageing. However, these overlapping genes exhibited high similarity in regulation and, as many of the other target genes were close to the threshold of a 1.3 fold-change, it is likely that many genes were missed out because of minor differences. In addition, the analysis of regulations in biological processes, cellular compartments, molecular functions and pathways showed that these were only in some cases overlapping or belonging to a similar cluster. These results indicate that the process of male ageing may differ from female ageing. However, the discussion on human skin ageing will be focussed on conserved mechanisms in both genders.

As expected, the overlapping genes were directly correlated with the observed overlap of biological processes, cellular compartments and Kegg pathways. Analysis of cellular compartments showed f.i. that genes hat contribute to the extracellular region and especially collagen-related genes such as *COL1A1*, *MATN4*, *TGFβ* and *MMP27* were down-regulated with age corresponding to previous findings (reviewed in [9]). Interestingly, dehydroepiandrosterone (DHEA), the most abundant human adrenal steroid, which exhibits significantly reduced levels with age, was shown to directly increase procollagen synthesis, and to inhibit collagen degradation [23]. Therefore, the decrease in extracellular structure especially through reduced collagen synthesis may be a direct effect of decreased hormone levels with age [8].

*ABCG1*, a member of the ATP-binding cassette transporters, responsible for macrophage cholesterol and phospholipid transport and ultimately for cellular lipid homeostasis [24] was downregulated with age. Analysis of biological processes revealed increased expression of cellular lipid catabolic processes especially in men. Furthermore, two lipid-related genes, *CPT1B* - a mitochondrial carnitine palmitoyltransferase required to allow net transport of long-chain fatty acyl-CoAs from the cytoplasm into the mitochondria for beta-oxidation [25] - and *FLJ20920* – an acyl-CoA synthetase- [26] validated the increased expression of lipid metabolism in males and females. As skin is getting dry with age, these results correspond to macroscopic findings [9].

Another conserved gene, which showed increased expression with age, is *ALDH4A1*. This gene has a p53-dependent protective role against cellular stress and especially overexpression of the gene showed significantly lower intracellular ROS levels after treatment with hydrogen peroxide or UV compared to controls [27]. Additionally, this gene is like the 2 above-mentioned up-regulated genes *CPT1B* and *FLJ2090* located in the mitochondria, the cellular compartment, which showed significantly increased regulation of related genes with age especially in females. These findings indicate an expected increase of ROS in aged skin compared to young.

Furthermore, in our data the sirtuin *SIRT6* showed increased expression in males and females with age. Sirtuins are the mammalian homologues of the yeast histone deacetylase Sir2. In recent years, emerging evidence indicates that these proteins (SIRT1-7) play key functions in cellular stress resistance, genomic stability, energy metabolism, ageing and tumourigenesis [28]. Among members of this family, SIRT6 appears to have particular significance in regulating metabolism, DNA repair and lifespan. Mice deficient in *SIRT6* exhibit shortened life span and a premature ageing-like phenotype [29]. Recently, the gene was proposed to contribute to the propagation of a specialised chromatin state at mammalian telomeres, which in turn is required for proper telomere metabolism and function [30]. Since telomere shortening is one of the mechanisms previously related to the ageing process, increased expression of this gene might be the cellular answer to shortened telomeres in old human skin.

Further interesting genes which were downregulated in our data with age were f.i. *LGR4*, a gene involved in the development of male genitalia and fertility [31] as well as epithelial cell proliferation [32], [33], [34] and *CORIN* (also known as atrial natriuretic peptide-converting enzyme), which is a cardiac protease that activates atrial natriuretic peptide (ANP), a cardiac hormone that is important in regulating blood pressure and promoting trophoblast invasion and spiral artery remodeling in pregnancy [34], [35].

In the first report describing the transcriptome of human sebocytes [8], we showed that genes involved in the pathogenesis of neurodegenerative diseases are expressed in this skin cell type and these genes are affected by the hormone environment. In order to justify the hypothesis, that skin may act as an adequate model of global ageing and especially of the nervous system- as both tissues are ectodermal derivatives [10] - we measured via RT-PCR amongst others the expression of *APP*, *TAU*, *PSEN1*, *PARK2-* which have been associated with Morbus Alzheimer and Morbus Parkinson, accordingly [18], [19], [20]. All genes showed to be expressed in human skin at mRNA level and particularly *APP* showed to be significantly downregulated in female aged skin, whereas *TAU* in males was significantly increased with age, in accordance to our array data. In addition, biological processes involved f.i. in neuron differentiation and development showed to be regulated in our data.

One of the conserved Kegg pathways was the WNT signalling pathway, which exhibited decreased regulation with age (Figure 2B, C). In addition to this, the GO-annotated biological processes WNT receptor signalling and the related frizzled signalling pathways were part of the conserved findings. The list of overlapping genes revealed 4 genes of WNT signalling pathway, which were downregulated with age amongst them *AXIN2*, a gene that modulates both amplitude and duration of active Wnt/β-catenin signalling pathway – mutations or loss of *AXIN2* have been associated with cancer and disturbance in the development of dentition [36], *FZD7* which acts as receptor for Wnt proteins and has been involved in carcinogenesis [37], *CPZ* – a metallocarboxypeptidase [38] and *WIF1*, a secreted Wnt antagonist. WNTs represent a large morphogenic family of secreted lipid-modified glycoproteins that control multiple developmental processes during embryogenesis [39] such as cell-fate specification, progenitor-cell proliferation [40] and the control of asymmetric cell division [41]. In addition, they have been associated in adult tissues and organs with tissue maintenance and remodelling and cancer progression [42]. Recently however, WNT signalling pathway has raised some interest in ageing research, although its main contribution to the ageing process remains unknown and controversially discussed [43], [44]. Inhibition of Wnt signalling may lead to a disruption of β-catenin signalling, thus leading β-catenin to activation of FOXO proteins and upregulation of genes coding for antioxidative enzymes. In addition, Wnt inhibition may lead to disinhibition of GSK-3, which in turn promotes TAU protein phosphorylation and may result in the formation of β-amyloid plaques (reviewed in [45]). This could explain the significant increase of *TAU* gene expression in the aged male donors (Figure 3). Furthermore, inhibition of Wnt signalling may lead to cytoskeletal changes, disturbances in adherence junction and cell adhesion [46].

Skin becomes with time xerotic, loses its anti-inflammatory properties and is prone to several infections and autoimmune diseases [9]. The role of the PPAR system in skin physiology has been thoroughly investigated in the last few years. This system is involved in skin cell proliferation, differentiation, apoptosis, inflammation and lipid synthesis- physiological processes which are hallmarks of aging [47], [48]. In contrast there is sparse data on Wnt signalling and skin. Recent studies have documented a cross-talk between Wnt and PPAR-δ signalling [49]. Our goal has been to understand the effects of a disturbed Wnt signalling on skin by investigating the consequences on the PPAR system. PPARs have been documented to be expressed in all compartments of the skin [50]. In consistence to our results, Nijsten et al 2005 proved that PPARα was downregulated in malignant and premalignant skin cancers, while PPARδ was upregulated [51]. PPARδ has been further linked to mitochondrial biogenesis, oxidative capacity and regulation of lipid oxidation. Overexpression of PPARδ in human cholangiocarcinoma cells has been shown to create a positive feed-forward loop that potentiates the actions of Wnt/β catenin signalling [52]. In our data overexpression of PPARδ may act as a counter weight to the downregulation of Wnt with age.

## Conclusions

In summary, the data on human skin ageing in both genders reveals some known age-related processes like the decrease in collagens and also some new insights like the possible involvement of WNT signalling. Furthermore, the potential regulation of different mechanisms including the loss of structure in the extracellular matrix, telomere shortening, and the involvement of sirtuins, highlight the complexity of the ageing process. Considering the increase in life expectancy, activities in the fields of preventive medicine and health support are needed to enable a longer time in good health. Understanding the mechanisms of ageing in humans of different gender can form the basis for comprehensive, knowledge-based prevention of age-related diseases and extension of healthy lifespan through the development of therapeutic products.

## Experimental Procedures

### Skin biopsies

Full-thickness skin biopsies were resected from the sun-protected inner side of the upper arm of volunteers. The Charité University Medicine Berlin ethics committee specifically approved this study. Ethical agreements were preliminarily obtained from all participants including written informed consent. Skin samples were provided by a total of 24 donors, all without any inflammatory or endocrinological disorders. The skin samples were obtained from European Caucasian young and elderly females (mean age 26.7±4 years [n1 = 7] and 70.75±3.3 years [n2 = 4], respectively) and males (mean age 25.8±5.2 years [n3 = 6] and 76±3.8 years [n4 = 7], respectively). One set of specimens was immediately soaked in RNAlater RNA Stabilization Reagent (Qiagen, Hilden, Germany) to prevent RNA degradation and was stored at 4°C for 24 h and then at -20°C until RNA isolation and microarray analysis (see RNA isolation and labelling) and another set was fixed in 10% formalin and embedded in paraffin.

### RNA extraction and reverse transcription

Human skin biopsies were homogenised in ∼400 µl of RLT buffer (Qiagen) using the Polytron PT3000 with a Polytron-Aggregate® homogenizer (Kinematika, Littau, Switzerland). The homogeniser was sequentially pre-treated with 3% H2O2 (Merck), 70% ethanol and dH2O to get rid of possible RNase contamination. Each sample was homogenised for 1 min then cooled on ice for 1 min and again homogenised for 1 min. After each sample the homogeniser was washed by treatment with dH2O, 70% ethanol and dH2O sequentially. RNA isolation from the homogenates was performed using the RNeasy® Mini Kit (Qiagen) including DNase I on column treatment following the manufacturer's protocol. The quantity of RNA and DNA was determined using the NanoDrop (NanoDrop Technologies, Wilmington, DE, USA). Agarose gel electrophoresis and ethidium bromide staining enabled the visualising of RNA and DNA for quality control. For reverse transcription Superscript II (Invitrogen, Life Technologies GmbH, Darmstadt, Germany) was used.

### Illumina bead chip hybridisation

Biotin-labelled cRNA was produced by means of a linear amplification kit (Ambion, Austin, TX, USA) using 300 ng of quality-checked total RNA as input. Chip hybridisations, washing, Cy3-streptavidin staining, and scanning were performed on an Illumina BeadStation 500 platform (Illumina, San Diego, CA, USA) using reagents and following protocols supplied by the manufacturer. cRNA samples were hybridised on human-8 (human skin biopsies) BeadChips. The following samples were hybridised in biological triplicate; synchronised young and aged human skin biopsies. All basic expression data analysis was carried out using the manufacturer's software BeadStudio 1.0 (Illumina). Raw data were background-subtracted and normalised using the ‘rank invariant’ algorithm, by which negative intensity values may arise. Normalised data were then filtered for significant expression on the basis of negative control beads. Selection for differentially expressed genes was performed on the basis of arbitrary thresholds for fold changes plus statistical significance according to an Illumina custom model [53].

### Pathway analysis

Differentially expressed genes were further filtered according to Gene Ontology (GO) terms or mapped to KEGG pathways using DAVID (http://david.abcc.ncifcrf.gov) [54].

For analysis GenBank accession numbers (Illumina data) represented by the corresponding chip oligonucleotides were used as input. Heatmaps were generated using “Multi Experiment Viewer” TM4 (TMEV.bat) [55]. WNT pathway related genes were obtained from the KEGG pathways analysis from the DAVID tool.

### Real-time polymerase chain reaction (Real-Time PCR) of selected genes

Real-Time PCR was performed as previously described [8] for following target genes: *TGFβ1* (f-ACCAAGAGAACGGAGCAGA, r-AGAGAC-TTTAGCCGCACCA), *AXIN2* (f-GCAATGGATTCAGGCAGAT, r-TCTTATGTAGG-TCTTG-GTGGC), *MIB1* (f-GGTCAATCGCCA-CTTGATC, r-ATAGAAGGAC-TCCGAGAACCC), *NLGN2* (f-CTGAGATCCTCATGCAGCA, r- GCCATACAGG-TTGTCCACA), *ATXN1* (f-AACAGGCCACTCATCGTGA, r-TGGTCTGAA-TGACCGTGTG), *WIF1* (Hs_WIF1_SG, QuantiTect Primer Assay (200) (QT00032298), Qiagen, Hilden, Germany), *SIRT6* (Hs_SIRT6_1_SG, QuantiTect Primer Assay (200) (QT00056812), Qiagen, Hilden, Germany), *B3GALT3* (Hs_B3GALNT1_1_SG QuantiTect Primer Assay, Qiagen, Hilden, Germany), *APP* (Hs_APP_2_SG QuantiTect Primer Assay (200) (QT01886815), Qiagen, Hilden, Germany), *TAU* (Hs_MAPT_1_SG QuantiTect Primer Assay (200) (QT00017199), Qiagen, Hilden, Germany), *PARK2* (Hs_PARK2_1_SG QuantiTect Primer Assay (200) (QT00023401), Qiagen, Hilden, Germany), *PSEN1* (Hs_PSEN1_1_SG QuantiTect Primer Assay (200) (QT00001862), Qiagen, Hilden, Germany). Triplicate amplifications were carried out per gene with three wells as negative controls without template. *PBGD* was amplified along with the target genes as endogenous controls for normalization. The PCR reaction was carried out on the ABI PRISM 7900HT Sequence Detection System (Applied Biosystems, Darmstadt, Germany). The output data generated by the Sequence Detection System 2 software were transferred to Excel (Microsoft, Redmond, WA, USA) for analysis. The differential mRNA expression of each gene was calculated with the comparative Ct (threshold cycle) method recommended by the manufacturer.

### Histology

Skin samples were fixed in 10% formalin and embedded in paraffin. Then they were sectioned and stained with haematoxylin/eosin and elastica staining for light microscopic observation. Morphometric analyses of images taken from light microscopy have been also used to determine the thickness of various skin layers in vitro. The epidermal thickness was measured from stratum basale to stratum granulosum (excluding stratum corneum), whereas the dermal thickness was the distance between the epidermis and the hypodermis.

### Immunohistochemistry

Immunohistochemistry was performed as previously described [56]. The preparations were incubated with the antibodies WIF-1 (sc-80453, mouse monoclonal), PPARδ (H-74, rabbit antibody) [all from Santa Cruz; Heidelberg, Germany] and FZD7 (ab-64636, rabbit polyclonal, Abcam, Cambridge, UK) at concentration of 1∶50, 1∶100 and 1∶700, respectively, at room temperature for 30 min, 24 hrs and 30 min, accordingly. All the antibodies were diluted with a background reducing antibody diluent (Dako, Hamburg, Germany). The secondary antibodies anti-mouse/anti-rabbit immunoglobulins (Dako) were used depending on the origin of the primary antibody. Diaminobenzidine (DAB) (Dako) (Figure 4 A–D) and LSAB, REAL Detection System (Dako) (Figure 4 E, F) visualization kits have been used. A semiquantitative method of immunohistochemical evaluation was used. Membrane or cytoplasmic staining of single cells was evaluated as positive. The intensity of the colour was objectively evaluated according to a 3 level scale: 0; not stained, 1; weak staining, 2; moderate staining, 3; intense staining. In addition, each of the skin structures was individually evaluated: epidermis, dermis, hair follicles, sebaceous ducts, sweat glands and sebaceous glands. The latter were further divided into basal, differentiating and mature cells. All slides were examined randomly and evaluated using the same scale. The procedure of staining was repeated twice for each case, each time individually evaluated. The XLSTAT programme was used. Statistical significance of the results of the immunohistological studies was calculated by the two-tailed Mann–Whitney test. Mean differences were considered to be significant at p<0.05.

## Supporting Information

Figure S1 **Global gene expression profiling of ageing in human skin biopsies from male and female donors.** Data reproducibility is demonstrated by sample correlation and clustering (A, B). Normalised data were analysed for significant (detection >0.99 for at least one group and p-value<0.05) changes in gene expression between young and old males and females with ratios of 1.3 and above. Venn diagram of gene expression in female and male aged vs. young skin- for the chosen criteria, there are more regulated genes in females (523) than in males (401) with age (C). In total, 39 genes are common in the target lists of significant regulated genes in males and females. The complete list of genes is given in Tables S1 and S2.
(TIF)

Table S1 **Complete list of genes regulated in female skin with age (** ***P*** **-value≤0.05; detection ratio 1.3).** Genes that were judged as non-detectable by the BG value criterion were excluded from analysis.
(XLS)

Table S2 **Complete list of genes regulated in male skin with age (** ***P*** **-value≤0.05; detection ratio 1.3).** Genes that were judged as non-detectable by the BG value criterion were excluded from analysis.
(XLS)

Table S3 **List of significantly (** ***P*** **-value<0.05) regulated GO Terms in female skin with age.** The enriched biological process (BP), cellular compartment (CC) and molecular function (MF) obtained by “DAVID Functional Annotation” is listed on the left side (Category; Term) and the corresponding number of regulated genes that were taken into account for computing the statistical test on the right (# genes) [% of the significantly regulated genes with age in females, accordingly; *P*-value; female up: upregulated processes with age, female down: downregulated processes with age].
(DOC)

Table S4 **List of significantly (** ***P*** **-value<0.05) regulated GO Terms in male skin with age.** The enriched biological process (BP), cellular compartment (CC) and molecular function (MF) obtained by “DAVID Functional Annotation” is listed on the left side (Category; Term) and the corresponding number of regulated genes that were taken into account for computing the statistical test on the right (# genes) [% of the significantly regulated genes with age in females, accordingly; *P*-value; male up: upregulated processes with age, male down: downregulated processes with age].
(DOC)

Table S5 **Results** **of the analysis of statistically significant pathways at the <0.1 level in skin biopsies of females with age.** Pathways were taken from the KEGG database (human pathways). Genes that were judged as non-detectable by the BG value criterion were excluded from analysis.
(DOC)

Table S6 **Results** **of the analysis of statistically significant pathways at the <0.1 level in skin biopsies of males with age.** Pathways were taken from the KEGG database (human pathways). Genes that were judged as non-detectable by the BG value criterion were excluded from analysis.
(DOC)

## References

[1] Smith JR, Pereira-Smith OM (1996) Replicative senescence: implications for in vivo aging and tumor suppression. Science 273: 63–67. 8658197 10.1126/science.273.5271.63

[2] Allsopp RC, Vaziri H, Patterson C, Goldstein S, Younglai EV, et al (1992) Telomere length predicts replicative capacity of human fibroblasts. Proc Natl Acad Sci U S A 89: 10114–10118. 1438199 10.1073/pnas.89.21.10114 PMC50288

[3] Michikawa Y, Mazzucchelli F, Bresolin N, Scarlato G, Attardi G (1999) Aging-dependent large accumulation of point mutations in the human mtDNA control region for replication. Science 286: 774–779. 10531063 10.1126/science.286.5440.774

[4] Miquel J (1998) An update on the oxygen stress-mitochondrial mutation theory of aging: genetic and evolutionary implications. Exp Gerontol 33: 113–126. 9467721 10.1016/s0531-5565(97)00060-0

[5] Ly DH, Lockhart DJ, Lerner RA, Schultz PG (2000) Mitotic misregulation and human aging. Science 287: 2486–2492. 10741968 10.1126/science.287.5462.2486

[6] Simon AF, Shih C, Mack A, Benzer S (2003) Steroid control of longevity in Drosophila melanogaster. Science 299: 1407–1410. 12610309 10.1126/science.1080539

[7] Tatar M, Bartke A, Antebi A (2003) The endocrine regulation of aging by insulin-like signals. Science 299: 1346–1351. 12610294 10.1126/science.1081447

[8] Makrantonaki E, Adjaye J, Herwig R, Brink TC, Groth D, et al (2006) Age-specific hormonal decline is accompanied by transcriptional changes in human sebocytes in vitro. Aging Cell 5: 331–344. 16805856 10.1111/j.1474-9726.2006.00223.x

[9] Zouboulis CC, Makrantonaki E (2011) Clinical aspects and molecular diagnostics of skin aging. Clin Dermatol 29: 3–14. 21146726 10.1016/j.clindermatol.2010.07.001

[10] Makrantonaki E, Schonknecht P, Hossini AM, Kaiser E, Katsouli MM, et al (2010) Skin and brain age together: The role of hormones in the ageing process. Exp Gerontol 45: 801–813. 20719245 10.1016/j.exger.2010.08.005

[11] Makrantonaki E, Zouboulis CC (2007) The skin as a mirror of the aging process in the human organism–state of the art and results of the aging research in the German National Genome Research Network 2 (NGFN-2). Exp Gerontol 42: 879–886. 17689905 10.1016/j.exger.2007.07.002

[12] Zahn JM, Poosala S, Owen AB, Ingram DK, Lustig A, et al (2007) AGEMAP: a gene expression database for aging in mice. PLoS Genet 3 11: e201. 18081424 10.1371/journal.pgen.0030201 PMC2098796

[13] Lener T, Moll PR, Rinnerthaler M, Bauer J, Aberger F, et al (2006) Expression profiling of aging in the human skin. Exp Gerontol 41: 387–397. 16530368 10.1016/j.exger.2006.01.012

[14] Capell BC, Tlougan BE, Orlow SJ (2009) From the rarest to the most common: insights from progeroid syndromes into skin cancer and aging. J Invest Dermatol 129: 2340–2350. 19387478 10.1038/jid.2009.103

[15] Gronniger E, Weber B, Heil O, Peters N, Stab F, et al (2010) Aging and chronic sun exposure cause distinct epigenetic changes in human skin. PLoS Genet 6: e1000971. 20523906 10.1371/journal.pgen.1000971 PMC2877750

[16] Renshaw M, Rockwell J, Engleman C, Gewirtz A, Katz J, et al (2002) Cutting edge: Impaired Toll ike receptor expression and function in aging. J Immunol 169: 4697–4701. 12391175 10.4049/jimmunol.169.9.4697

[17] Linford NJ, Beyer R, Gollahon K, Krajcik RA, Malloy VL, et al (2007) Transcriptional response to aging and caloric restriction in heart and adipose tissue. Aging Cell 6: 673–688. 17874999 10.1111/j.1474-9726.2007.00319.x

[18] Murrell J, Farlow M, Ghetti B, Benson MD (1991) A mutation in the amyloid precursor protein associated with hereditary Alzheimer's disease. Science 254: 97–99. 1925564 10.1126/science.1925564

[19] Devi G, Fotiou A, Jyrinji D, Tycko B, DeArmand S, et al (2000) Novel presenilin 1 mutations associated with early onset of dementia in a family with both early-onset and late-onset Alzheimer disease. Arch Neurol 57: 1454–1457. 11030797 10.1001/archneur.57.10.1454

[20] Matsumine H, Saito M, Shimoda-Matsubayashi S, Tanaka H, Ishikawa A, et al (1997) Localization of a gene for an autosomal recessive form of juvenile Parkinsonism to chromosome 6q25.2-27. Am J Hum Genet 60: 588–596. 9042918 PMC1712507

[21] Azzi L, El-Alfy M, Martel C, Labrie F (2005) Gender differences in mouse skin morphology and specific effects of sex steroids and dehydroepiandrosterone. J Invest Dermatol 124: 22–27. 15654949 10.1111/j.0022-202X.2004.23545.x

[22] Seidenari S, Pagnoni A, Di Nardo A, Giannetti A (1994) Echographic evaluation with image analysis of normal skin: variations according to age and sex. Skin Pharmacol 7: 201–209. 8024801 10.1159/000211295

[23] Shin MH, Rhie GE, Park CH, Kim KH, Cho KH, et al (2005) Modulation of collagen metabolism by the topical application of dehydroepiandrosterone to human skin. J Invest Dermatol 124: 315–323. 15675949 10.1111/j.0022-202X.2004.23588.x

[24] Baldan A, Tarr P, Lee R, Edwards PA (2006) ATP-binding cassette transporter G1 and lipid homeostasis. Curr Opin Lipidol 17: 227–232. 16680026 10.1097/01.mol.0000226113.89812.bb

[25] Yamazaki N, Shinohara Y, Shima A, Yamanaka Y, Terada H (1996) Isolation and characterization of cDNA and genomic clones encoding human muscle type carnitine palmitoyltransferase I. . Biochim Biophys Acta 1307: 157–161. 8679700 10.1016/0167-4781(96)00069-3

[26] Watkins PA, Maiguel D, Jia Z, Pevsner J (2007) Evidence for 26 distinct acyl-coenzyme A synthetase genes in the human genome. J Lipid Res 48: 2736–2750. 17762044 10.1194/jlr.M700378-JLR200

[27] Yoon KA, Nakamura Y, Arakawa H (2004) Identification of ALDH4 as a p53-inducible gene and its protective role in cellular stresses. J Hum Genet 49: 134–140. 14986171 10.1007/s10038-003-0122-3

[28] Zhong L, Mostoslavsky R (2010) SIRT6: A master epigenetic gatekeeper of glucose metabolism. Transcr 1: 17–21. 10.4161/trns.1.1.12143 PMC3035182 21327158

[29] Mostoslavsky R, Chua KF, Lombard DB, Pang WW, Fischer MR, et al (2006) Genomic instability and aging-like phenotype in the absence of mammalian SIRT6. Cell 124: 315–329. 16439206 10.1016/j.cell.2005.11.044

[30] Michishita E, McCord RA, Berber E, Kioi M, Padilla-Nash H, et al (2008) SIRT6 is a histone H3 lysine 9 deacetylase that modulates telomeric chromatin. Nature 452: 492–496. 18337721 10.1038/nature06736 PMC2646112

[31] Hoshii T, Takeo T, Nakagata N, Takeya M, Araki K, et al (2007) LGR4 regulates the postnatal development and integrity of male reproductive tracts in mice. Biol Reprod 76 2: 303–131. 17079737 10.1095/biolreprod.106.054619

[32] Oyama K, Mohri Y, Sone M, Nawa A, Nishimori K (2011) Conditional knockout of Lgr4 leads to impaired ductal elongation and branching morphogenesis in mouse mammary glands. Sex Dev 5 4: 205–12. 21791950 10.1159/000329476

[33] Mustata RC, Van Loy T, Lefort A, Libert F, Strollo S, et al (2011) Lgr4 is required for Paneth cell differentiation and maintenance of intestinal stem cells ex vivo. EMBO Rep 12 6: 558–64. 21508962 10.1038/embor.2011.52 PMC3128273

[34] Wu Q, Xu-Cai YO, Chen S, Wang W (2009) Corin: new insights into the natriuretic peptide system. Kidney Int 75 2: 142–6. 18716601 10.1038/ki.2008.418 PMC2655200

[35] Cui Y, Wang W, Dong N, Lou J, Srinivasan DK, et al (2012) Role of corin in trophoblast invasion and uterine spiral artery remodeling in pregnancy. Nature 484 7393: 246–50. 22437503 10.1038/nature10897 PMC3578422

[36] Lammi L, Arte S, Somer M, Jarvinen H, Lahermo P, et al (2004) Mutations in AXIN2 cause familial tooth agenesis and predispose to colorectal cancer. Am J Hum Genet 74: 1043–1050. 15042511 10.1086/386293 PMC1181967

[37] Wei W, Chua M, Grepper S, So S (2011) Soluble Frizzled-7 receptor inhibits Wnt signaling and sensitizes hepatocellular carcinoma cells towards doxorubicin. Mol Cancer 10: 16. 21314951 10.1186/1476-4598-10-16 PMC3050858

[38] Song L, Fricker LD (1997) Cloning and expression of human carboxypeptidase Z, a novel metallocarboxypeptidase. J Biol Chem 272: 10543–10550. 9099699 10.1074/jbc.272.16.10543

[39] Dreesen O, Brivanlou AH (2007) Signaling pathways in cancer and embryonic stem cells. Stem Cell Rev 3: 7–17. 17873377 10.1007/s12015-007-0004-8

[40] Adjaye J, Huntriss J, Herwig R, BenKahla A, Brink TC, et al (2005) Primary differentiation in the human blastocyst: comparative molecular portraits of inner cell mass and trophectoderm cells. Stem Cells 23: 1514–1525. 16081659 10.1634/stemcells.2005-0113

[41] Sethi JK, Vidal-Puig A (2010) A Wnt signalling and the control of cellular metabolism. Biochem J 427: 1–17. 20226003 10.1042/BJ20091866 PMC4301310

[42] van den Brink GR, Hardwick JC (2006) Hedgehog Wnt interaction in colorectal cancer. Gut 55: 912–914. 16766747 10.1136/gut.2005.085902 PMC1856303

[43] Carlson ME, Silva HS, Conboy IM (2008) Aging of signal transduction pathways, and pathology. Exp Cell Res 314: 1951–1961. 18474281 10.1016/j.yexcr.2008.03.017 PMC2572856

[44] DeCarolis NA, Wharton KA Jr, Eisch AJ (2008) Which way does the Wnt blow? Exploring the duality of canonical Wnt signaling on cellular aging. Bioessays 30: 102–106. 18200563 10.1002/bies.20709

[45] Manolopoulos KN, Klotz LO, Korsten P, Bornstein SR, Barthel A (2010) Linking Alzheimer's disease to insulin resistance: the FoxO response to oxidative stress. . Mol Psychiatry 11: 1046–52. 20966918 10.1038/mp.2010.17

[46] Heuberger J, Birchmeier W (2010) Interplay of Cadherin-Mediated Cell Adhesion and Canonical Wnt Signaling. Cold Spring Harb Perspect Biol 2: a002915. 20182623 10.1101/cshperspect.a002915 PMC2828280

[47] Michalik L, Wahli W (2007) Peroxisome proliferator-activated receptors (PPARs) in skin health, repair and disease. Biochim Biophys Acta 1771: 991–998. 17400022 10.1016/j.bbalip.2007.02.004

[48] Makrantonaki E, Zouboulis CC (2007) Testosterone metabolism to 5alpha-dihydrotestosterone and synthesis of sebaceous lipids is regulated by the peroxisome proliferator-activated receptor ligand linoleic acid in human sebocytes. Br J Dermatol 156: 428–432. 17300229 10.1111/j.1365-2133.2006.07671.x

[49] Lemay DG, Hwang DH (2006) Genome-wide identification of peroxisome proliferator response elements using integrated computational genomics. J Lipid Res 47: 1583–1587. 16585784 10.1194/jlr.M500504-JLR200

[50] Chen W, Yang CC, Sheu HM, Seltmann H, Zouboulis CC (2003) Expression of peroxisome proliferator-activated receptor and CCAAT/enhancer binding protein transcription factors in cultured human sebocytes. J Invest Dermatol 121: 441–447. 12925198 10.1046/j.1523-1747.2003.12411.x

[51] Nijsten T, Geluyckens E, Colpaert C, Lambert J (2005) Peroxisome proliferator-activated receptors in squamous cell carcinoma and its precursors. J Cutan Pathol 32: 340–347. 15811118 10.1111/j.0303-6987.2005.00345.x

[52] Han C, Lim K, Xu L, Li G, Wu T (2008) Regulation of Wnt/beta-catenin pathway by cPLA2alpha and PPARdelta. J Cell Biochem 105: 534–545. 18636547 10.1002/jcb.21852 PMC2593467

[53] Kuhn K, Baker SC, Chudin E, Lieu MH, Oeser S, et al (2004) A novel, high-performance random array platform for quantitative gene expression profiling. Genome Res 14 11: 2347–56. 15520296 10.1101/gr.2739104 PMC525694

[54] Huang da W, Sherman BT, Lempicki RA (2009) Systematic and integrative analysis of large gene lists using DAVID bioinformatics resources. Nat Protoc 4 1: 44–57. 19131956 10.1038/nprot.2008.211

[55] Saeed AI, Sharov V, White J, Li J, Liang W, et al (2003) TM4: a free, open-source system for microarray data management and analysis. Biotechniques 34 2: 374–8. 12613259 10.2144/03342mt01

[56] Zouboulis CC, Seltmann H, Hiroi N, Chen W, Young M, et al (2002) Corticotropin-releasing hormone: an autocrine hormone that promotes lipogenesis in human sebocytes. Proc Natl Acad Sci U S A 99: 7148–7153. 12011471 10.1073/pnas.102180999 PMC124543

