# Supplementary figures and images for: Identification of Biomarkers of Human Skin Ageing in Both Genders. Wnt Signalling - A Label of Skin Ageing?
Source: PLoS One. 2012 Nov 30;7(11):e50393. doi: 10.1371/journal.pone.0050393 (PMC3511529; doi:10.1371/journal.pone.0050393)

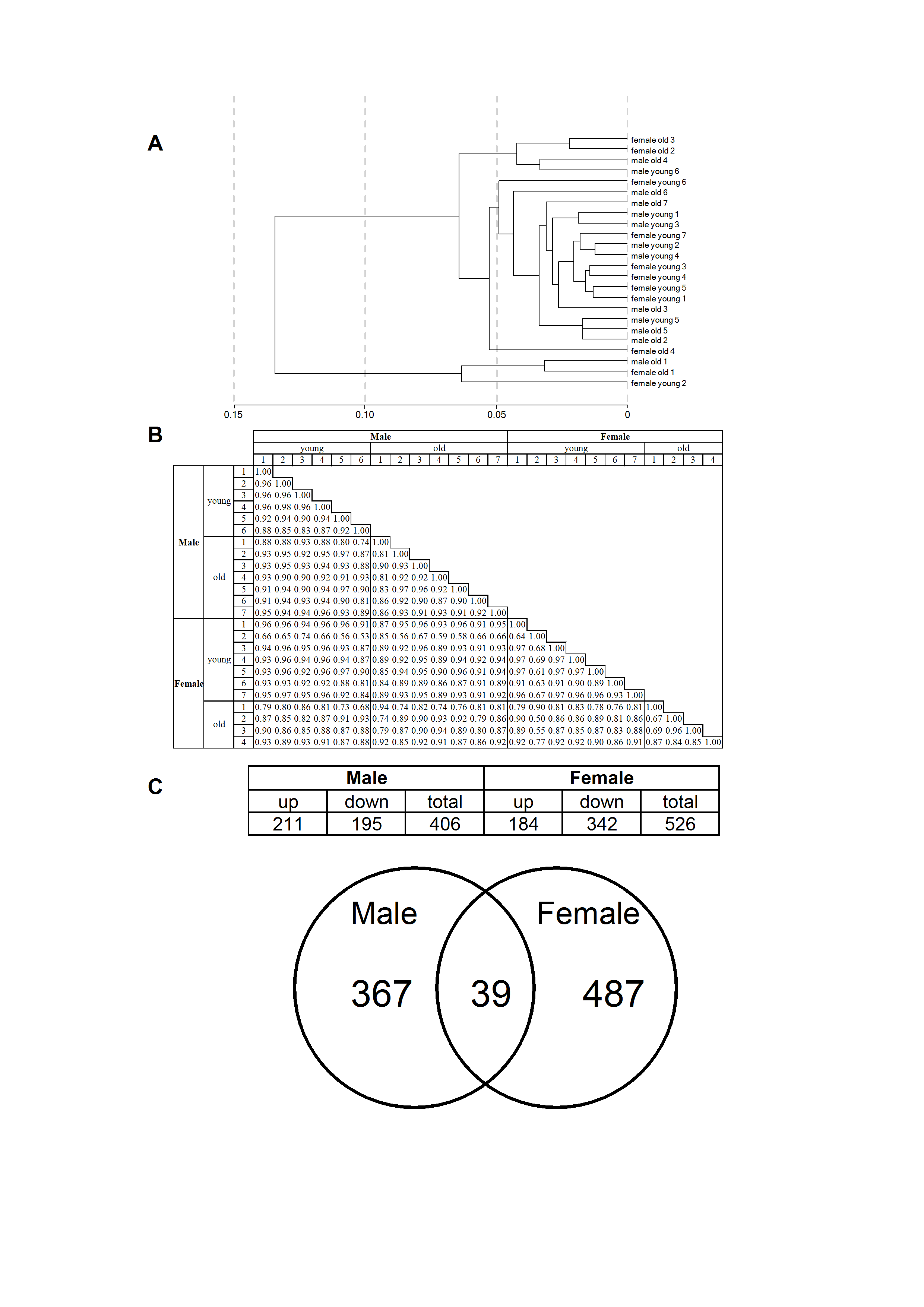

Supplement: Figure S1 — Global gene expression profiling of ageing in human skin biopsies from male and female donors. Data reproducibility is demonstrated by sample correlation and clustering (A, B). Normalised data were analysed for significant (detection >0.99 for at least one group and p-value<0.05) changes in gene expression between young and old males and females with ratios of 1.3 and above. Venn diagram of gene expression in female and male aged vs. young skin- for the chosen criteria, there are more regulated genes in females (523) than in males (401) with age (C). In total, 39 genes are common in the target lists of significant regulated genes in males and females. The complete list of genes is given in Tables S1 and S2. (TIF) [file pone.0050393.s001.tif]
