# Supplementary material for: Identification of Biomarkers of Human Skin Ageing in Both Genders. Wnt Signalling - A Label of Skin Ageing?
Source: PLoS One. 2012 Nov 30;7(11):e50393. doi: 10.1371/journal.pone.0050393 (PMC3511529; doi:10.1371/journal.pone.0050393)
Supplement: Table S3 — List of significantly ( P -value<0.05) regulated GO Terms in female skin with age. The enriched biological process (BP), cellular compartment (CC) and molecular function (MF) obtained by “DAVID Functional Annotation” is listed on the left side (Category; Term) and the corresponding number of regulated genes that were taken into account for computing the statistical test on the right (# genes) [% of the significantly regulated genes with age in females, accordingly; P-value; female up: upregulated processes with age, female down: downregulated processes with age]. (DOC) [file pone.0050393.s004.doc]

| **Category** | **Term** | **Count** | **%** | ***P*-Value** |
| --- | --- | --- | --- | --- |
| **Female Up** |  |  |  |  |
| **GOTERM_BP_FAT** |  |  |  |  |
| GO:0006412 | translation | 11 | 6.011 | 0.001 |
| GO:0043039 | tRNA aminoacylation | 4 | 2.186 | 0.010 |
| GO:0006418 | tRNA aminoacylation for protein translation | 4 | 2.186 | 0.010 |
| GO:0043038 | amino acid activation | 4 | 2.186 | 0.010 |
| GO:0034660 | ncRNA metabolic process | 7 | 3.825 | 0.025 |
| GO:0006399 | tRNA metabolic process | 5 | 2.732 | 0.028 |
| GO:0006419 | alanyl-tRNA aminoacylation | 2 | 1.093 | 0.029 |
| GO:0009267 | cellular response to starvation | 3 | 1.639 | 0.039 |
| GO:0006396 | RNA processing | 11 | 6.011 | 0.041 |
| **GOTERM_CC_FAT** |  |  |  |  |
| GO:0005739 | mitochondrion | 24 | 13.115 | 0.000 |
| GO:0030286 | dynein complex | 4 | 2.186 | 0.004 |
| GO:0000313 | organellar ribosome | 4 | 2.186 | 0.010 |
| GO:0005761 | mitochondrial ribosome | 4 | 2.186 | 0.010 |
| GO:0005762 | mitochondrial large ribosomal subunit | 3 | 1.639 | 0.012 |
| GO:0000315 | organellar large ribosomal subunit | 3 | 1.639 | 0.012 |
| GO:0005875 | microtubule associated complex | 5 | 2.732 | 0.015 |
| GO:0005759 | mitochondrial matrix | 7 | 3.825 | 0.019 |
| GO:0031980 | mitochondrial lumen | 7 | 3.825 | 0.019 |
| GO:0005829 | cytosol | 21 | 11.475 | 0.021 |
| GO:0035085 | cilium axoneme | 3 | 1.639 | 0.022 |
| GO:0044429 | mitochondrial part | 12 | 6.557 | 0.023 |
| GO:0005840 | ribosome | 6 | 3.279 | 0.050 |
| **GOTERM_MF_FAT** |  |  |  |  |
| GO:0004812 | aminoacyl-tRNA ligase activity | 4 | 2.186 | 0.011 |
| GO:0016875 | ligase activity. forming carbon-oxygen bonds | 4 | 2.186 | 0.011 |
| GO:0016876 | ligase activity. forming aminoacyl-tRNA and related compounds | 4 | 2.186 | 0.011 |
| GO:0008092 | cytoskeletal protein binding | 12 | 6.557 | 0.012 |
| GO:0004813 | alanine-tRNA ligase activity | 2 | 1.093 | 0.030 |
| GO:0005524 | ATP binding | 23 | 12.568 | 0.034 |
| GO:0032555 | purine ribonucleotide binding | 27 | 14.754 | 0.037 |
| GO:0032553 | ribonucleotide binding | 27 | 14.754 | 0.037 |
| GO:0032559 | adenyl ribonucleotide binding | 23 | 12.568 | 0.038 |
| GO:0008599 | protein phosphatase type 1 regulator activity | 2 | 1.093 | 0.039 |
| GO:0000166 | nucleotide binding | 31 | 16.940 | 0.050 |
|  |  |  |  |  |
| **Female down** |  |  |  |  |
| **GOTERM_BP_FAT** |  |  |  |  |
| GO:0006414 | translational elongation | 34 | 10.029 | 0.000 |
| GO:0006412 | translation | 35 | 10.324 | 0.000 |
| GO:0030198 | extracellular matrix organization | 19 | 5.605 | 0.000 |
| GO:0043062 | extracellular structure organization | 21 | 6.195 | 0.000 |
| GO:0001501 | skeletal system development | 23 | 6.785 | 0.000 |
| GO:0042274 | ribosomal small subunit biogenesis | 6 | 1.770 | 0.000 |
| GO:0001503 | ossification | 12 | 3.540 | 0.000 |
| GO:0060348 | bone development | 12 | 3.540 | 0.000 |
| GO:0007155 | cell adhesion | 31 | 9.145 | 0.000 |
| GO:0022610 | biological adhesion | 31 | 9.145 | 0.000 |
| GO:0001957 | intramembranous ossification | 4 | 1.180 | 0.000 |
| GO:0006364 | rRNA processing | 9 | 2.655 | 0.000 |
| GO:0016072 | rRNA metabolic process | 9 | 2.655 | 0.001 |
| GO:0042254 | ribosome biogenesis | 10 | 2.950 | 0.001 |
| GO:0045935 | positive regulation of nucleobase. nucleoside. nucleotide and nucleic acid metabolic process | 25 | 7.375 | 0.001 |
| GO:0048705 | skeletal system morphogenesis | 9 | 2.655 | 0.002 |
| GO:0003002 | regionalization | 12 | 3.540 | 0.002 |
| GO:0009952 | anterior/posterior pattern formation | 10 | 2.950 | 0.002 |
| GO:0051173 | positive regulation of nitrogen compound metabolic process | 25 | 7.375 | 0.002 |
| GO:0032963 | collagen metabolic process | 5 | 1.475 | 0.002 |
| GO:0030199 | collagen fibril organization | 5 | 1.475 | 0.002 |
| GO:0009891 | positive regulation of biosynthetic process | 26 | 7.670 | 0.002 |
| GO:0044259 | multicellular organismal macromolecule metabolic process | 5 | 1.475 | 0.003 |
| GO:0051216 | cartilage development | 7 | 2.065 | 0.003 |
| GO:0007507 | heart development | 12 | 3.540 | 0.003 |
| GO:0045941 | positive regulation of transcription | 22 | 6.490 | 0.003 |
| GO:0032964 | collagen biosynthetic process | 3 | 0.885 | 0.004 |
| GO:0031328 | positive regulation of cellular biosynthetic process | 25 | 7.375 | 0.004 |
| GO:0010557 | positive regulation of macromolecule biosynthetic process | 24 | 7.080 | 0.004 |
| GO:0016055 | Wnt receptor signaling pathway | 9 | 2.655 | 0.004 |
| GO:0010628 | positive regulation of gene expression | 22 | 6.490 | 0.005 |
| GO:0044236 | multicellular organismal metabolic process | 5 | 1.475 | 0.006 |
| GO:0007389 | pattern specification process | 13 | 3.835 | 0.006 |
| GO:0022613 | ribonucleoprotein complex biogenesis | 10 | 2.950 | 0.009 |
| GO:0006029 | proteoglycan metabolic process | 5 | 1.475 | 0.010 |
| GO:0010324 | membrane invagination | 11 | 3.245 | 0.011 |
| GO:0006897 | endocytosis | 11 | 3.245 | 0.011 |
| GO:0030182 | neuron differentiation | 17 | 5.015 | 0.012 |
| GO:0045893 | positive regulation of transcription. DNA-dependent | 18 | 5.310 | 0.012 |
| GO:0060325 | face morphogenesis | 3 | 0.885 | 0.012 |
| GO:0051254 | positive regulation of RNA metabolic process | 18 | 5.310 | 0.013 |
| GO:0034101 | erythrocyte homeostasis | 5 | 1.475 | 0.015 |
| GO:0042273 | ribosomal large subunit biogenesis | 3 | 0.885 | 0.015 |
| GO:0048666 | neuron development | 14 | 4.130 | 0.015 |
| GO:0010604 | positive regulation of macromolecule metabolic process | 27 | 7.965 | 0.016 |
| GO:0043588 | skin development | 4 | 1.180 | 0.018 |
| GO:0060323 | head morphogenesis | 3 | 0.885 | 0.018 |
| GO:0060324 | face development | 3 | 0.885 | 0.018 |
| GO:0042476 | odontogenesis | 5 | 1.475 | 0.021 |
| GO:0002683 | negative regulation of immune system process | 6 | 1.770 | 0.023 |
| GO:0060021 | palate development | 4 | 1.180 | 0.024 |
| GO:0001756 | somitogenesis | 4 | 1.180 | 0.028 |
| GO:0031175 | neuron projection development | 11 | 3.245 | 0.028 |
| GO:0016567 | protein ubiquitination | 7 | 2.065 | 0.029 |
| GO:0010171 | body morphogenesis | 3 | 0.885 | 0.029 |
| GO:0060322 | head development | 3 | 0.885 | 0.029 |
| GO:0045944 | positive regulation of transcription from RNA polymerase II promoter | 14 | 4.130 | 0.029 |
| GO:0034470 | ncRNA processing | 9 | 2.655 | 0.030 |
| GO:0006984 | ER-nuclear signaling pathway | 4 | 1.180 | 0.030 |
| GO:0042127 | regulation of cell proliferation | 24 | 7.080 | 0.033 |
| GO:0008203 | cholesterol metabolic process | 6 | 1.770 | 0.034 |
| GO:0040008 | regulation of growth | 13 | 3.835 | 0.035 |
| GO:0042592 | homeostatic process | 23 | 6.785 | 0.036 |
| GO:0060346 | bone trabecula formation | 2 | 0.590 | 0.039 |
| GO:0046489 | phosphoinositide biosynthetic process | 4 | 1.180 | 0.042 |
| GO:0002684 | positive regulation of immune system process | 10 | 2.950 | 0.043 |
| GO:0046474 | glycerophospholipid biosynthetic process | 5 | 1.475 | 0.043 |
| GO:0032446 | protein modification by small protein conjugation | 7 | 2.065 | 0.044 |
| GO:0045449 | regulation of transcription | 63 | 18.584 | 0.045 |
| GO:0048592 | eye morphogenesis | 5 | 1.475 | 0.045 |
| GO:0050654 | chondroitin sulfate proteoglycan metabolic process | 3 | 0.885 | 0.047 |
| GO:0016125 | sterol metabolic process | 6 | 1.770 | 0.047 |
| GO:0001649 | osteoblast differentiation | 4 | 1.180 | 0.048 |
| GO:0050868 | negative regulation of T cell activation | 4 | 1.180 | 0.048 |
| GO:0000904 | cell morphogenesis involved in differentiation | 10 | 2.950 | 0.049 |
| GO:0001568 | blood vessel development | 10 | 2.950 | 0.049 |
| GO:0030218 | erythrocyte differentiation | 4 | 1.180 | 0.051 |
| **GOTERM_CC_FAT** |  |  |  |  |
| GO:0022626 | cytosolic ribosome | 31 | 9.145 | 0.000 |
| GO:0033279 | ribosomal subunit | 31 | 9.145 | 0.000 |
| GO:0022627 | cytosolic small ribosomal subunit | 20 | 5.900 | 0.000 |
| GO:0044445 | cytosolic part | 31 | 9.145 | 0.000 |
| GO:0005840 | ribosome | 33 | 9.735 | 0.000 |
| GO:0015935 | small ribosomal subunit | 20 | 5.900 | 0.000 |
| GO:0031012 | extracellular matrix | 31 | 9.145 | 0.000 |
| GO:0005578 | proteinaceous extracellular matrix | 28 | 8.260 | 0.000 |
| GO:0022625 | cytosolic large ribosomal subunit | 11 | 3.245 | 0.000 |
| GO:0030529 | ribonucleoprotein complex | 34 | 10.029 | 0.000 |
| GO:0015934 | large ribosomal subunit | 11 | 3.245 | 0.000 |
| GO:0044421 | extracellular region part | 43 | 12.684 | 0.000 |
| GO:0005576 | extracellular region | 67 | 19.764 | 0.000 |
| GO:0005581 | collagen | 7 | 2.065 | 0.000 |
| GO:0005829 | cytosol | 48 | 14.159 | 0.000 |
| GO:0044420 | extracellular matrix part | 10 | 2.950 | 0.001 |
| GO:0005583 | fibrillar collagen | 4 | 1.180 | 0.002 |
| GO:0005614 | interstitial matrix | 3 | 0.885 | 0.017 |
| GO:0031091 | platelet alpha granule | 5 | 1.475 | 0.029 |
| GO:0043228 | non-membrane-bounded organelle | 67 | 19.764 | 0.041 |
| GO:0043232 | intracellular non-membrane-bounded organelle | 67 | 19.764 | 0.041 |
| GO:0005584 | collagen type I | 2 | 0.590 | 0.041 |
| **GOTERM_MF_FAT** |  |  |  |  |
| GO:0003735 | structural constituent of ribosome | 31 | 9.145 | 0.000 |
| GO:0005198 | structural molecule activity | 43 | 12.684 | 0.000 |
| GO:0019838 | growth factor binding | 11 | 3.245 | 0.000 |
| GO:0048407 | platelet-derived growth factor binding | 5 | 1.475 | 0.000 |
| GO:0003723 | RNA binding | 31 | 9.145 | 0.000 |
| GO:0005201 | extracellular matrix structural constituent | 9 | 2.655 | 0.000 |
| GO:0003700 | transcription factor activity | 34 | 10.029 | 0.001 |
| GO:0046332 | SMAD binding | 6 | 1.770 | 0.002 |
| GO:0005518 | collagen binding | 5 | 1.475 | 0.005 |
| GO:0005520 | insulin-like growth factor binding | 4 | 1.180 | 0.013 |
| GO:0030528 | transcription regulator activity | 43 | 12.684 | 0.013 |
| GO:0003727 | single-stranded RNA binding | 4 | 1.180 | 0.015 |
| GO:0019843 | rRNA binding | 4 | 1.180 | 0.019 |
| GO:0008239 | dipeptidyl-peptidase activity | 3 | 0.885 | 0.019 |
| GO:0008236 | serine-type peptidase activity | 9 | 2.655 | 0.024 |
| GO:0017171 | serine hydrolase activity | 9 | 2.655 | 0.026 |
| GO:0043565 | sequence-specific DNA binding | 20 | 5.900 | 0.031 |
| GO:0016563 | transcription activator activity | 15 | 4.425 | 0.032 |
| GO:0004175 | endopeptidase activity | 14 | 4.130 | 0.034 |
| GO:0030674 | protein binding. bridging | 6 | 1.770 | 0.038 |
| GO:0004287 | prolyl oligopeptidase activity | 2 | 0.590 | 0.039 |
