# Supplementary material for: Identification of Biomarkers of Human Skin Ageing in Both Genders. Wnt Signalling - A Label of Skin Ageing?
Source: PLoS One. 2012 Nov 30;7(11):e50393. doi: 10.1371/journal.pone.0050393 (PMC3511529; doi:10.1371/journal.pone.0050393)
Supplement: Table S4 — List of significantly ( P -value<0.05) regulated GO Terms in male skin with age. The enriched biological process (BP), cellular compartment (CC) and molecular function (MF) obtained by “DAVID Functional Annotation” is listed on the left side (Category; Term) and the corresponding number of regulated genes that were taken into account for computing the statistical test on the right (# genes) [% of the significantly regulated genes with age in females, accordingly; P-value; male up: upregulated processes with age, male down: downregulated processes with age]. (DOC) [file pone.0050393.s005.doc]

| **Category** | **Term** | **Count** | **%** | ***P*-Value** |
| --- | --- | --- | --- | --- |
| **Male Up** |  |  |  |  |
| **GOTERM_BP_FAT** |  |  |  |  |
| GO:0030162 | regulation of proteolysis | 5 | 2.381 | 0.003 |
| GO:0046942 | carboxylic acid transport | 7 | 3.333 | 0.006 |
| GO:0015849 | organic acid transport | 7 | 3.333 | 0.006 |
| GO:0006644 | phospholipid metabolic process | 7 | 3.333 | 0.018 |
| GO:0030163 | protein catabolic process | 14 | 6.667 | 0.019 |
| GO:0006944 | membrane fusion | 4 | 1.905 | 0.020 |
| GO:0048284 | organelle fusion | 3 | 1.429 | 0.022 |
| GO:0019637 | organophosphate metabolic process | 7 | 3.333 | 0.023 |
| GO:0009057 | macromolecule catabolic process | 16 | 7.619 | 0.024 |
| GO:0044265 | cellular macromolecule catabolic process | 15 | 7.143 | 0.028 |
| GO:0002889 | regulation of immunoglobulin mediated immune response | 3 | 1.429 | 0.028 |
| GO:0051180 | vitamin transport | 3 | 1.429 | 0.028 |
| GO:0002712 | regulation of B cell mediated immunity | 3 | 1.429 | 0.028 |
| GO:0060249 | anatomical structure homeostasis | 5 | 2.381 | 0.029 |
| GO:0045862 | positive regulation of proteolysis | 3 | 1.429 | 0.030 |
| GO:0006687 | glycosphingolipid metabolic process | 3 | 1.429 | 0.033 |
| GO:0000018 | regulation of DNA recombination | 3 | 1.429 | 0.033 |
| GO:0051052 | regulation of DNA metabolic process | 5 | 2.381 | 0.036 |
| GO:0006635 | fatty acid beta-oxidation | 3 | 1.429 | 0.037 |
| GO:0046474 | glycerophospholipid biosynthetic process | 4 | 1.905 | 0.038 |
| GO:0016042 | lipid catabolic process | 6 | 2.857 | 0.041 |
| GO:0007042 | lysosomal lumen acidification | 2 | 0.952 | 0.043 |
| GO:0006664 | glycolipid metabolic process | 3 | 1.429 | 0.045 |
| GO:0006665 | sphingolipid metabolic process | 4 | 1.905 | 0.049 |
| GO:0044242 | cellular lipid catabolic process | 4 | 1.905 | 0.051 |
| **GOTERM_CC_FAT** |  |  |  |  |
| GO:0000267 | cell fraction | 22 | 10.476 | 0.005 |
| GO:0005724 | nuclear telomeric heterochromatin | 2 | 0.952 | 0.021 |
| GO:0031933 | telomeric heterochromatin | 2 | 0.952 | 0.021 |
| GO:0005624 | membrane fraction | 16 | 7.619 | 0.024 |
| GO:0005626 | insoluble fraction | 16 | 7.619 | 0.031 |
| GO:0042599 | lamellar body | 2 | 0.952 | 0.042 |
| GO:0044456 | synapse part | 7 | 3.333 | 0.046 |
| GO:0008021 | synaptic vesicle | 4 | 1.905 | 0.046 |
| GO:0005625 | soluble fraction | 8 | 3.810 | 0.048 |
| **GOTERM_MF_FAT** |  |  |  |  |
| GO:0016645 | oxidoreductase activity. acting on the CH-NH group of donors | 4 | 1.905 | 0.003 |
| GO:0016646 | oxidoreductase activity. acting on the CH-NH group of donors. NAD or NADP as acceptor | 3 | 1.429 | 0.015 |
| GO:0016563 | transcription activator activity | 10 | 4.762 | 0.028 |
| GO:0042802 | identical protein binding | 13 | 6.190 | 0.036 |
| GO:0004674 | protein serine/threonine kinase activity | 10 | 4.762 | 0.037 |
| **Male down** |  |  |  |  |
| **GOTERM_BP_FAT** |  |  |  |  |
| GO:0048066 | pigmentation during development | 5 | 2.632 | 0.000 |
| GO:0016055 | Wnt receptor signaling pathway | 8 | 4.211 | 0.000 |
| GO:0042438 | melanin biosynthetic process | 3 | 1.579 | 0.003 |
| GO:0043473 | pigmentation | 5 | 2.632 | 0.003 |
| GO:0006582 | melanin metabolic process | 3 | 1.579 | 0.004 |
| GO:0045449 | regulation of transcription | 39 | 20.526 | 0.012 |
| GO:0030514 | negative regulation of BMP signaling pathway | 3 | 1.579 | 0.019 |
| GO:0006471 | protein amino acid ADP-ribosylation | 3 | 1.579 | 0.021 |
| GO:0006355 | regulation of transcription. DNA-dependent | 28 | 14.737 | 0.022 |
| GO:0043954 | cellular component maintenance | 3 | 1.579 | 0.027 |
| GO:0051252 | regulation of RNA metabolic process | 28 | 14.737 | 0.029 |
| GO:0030510 | regulation of BMP signaling pathway | 3 | 1.579 | 0.038 |
| GO:0042441 | eye pigment metabolic process | 2 | 1.053 | 0.040 |
| GO:0048069 | eye pigmentation | 2 | 1.053 | 0.040 |
| GO:0034331 | cell junction maintenance | 2 | 1.053 | 0.040 |
| GO:0045217 | cell-cell junction maintenance | 2 | 1.053 | 0.040 |
| GO:0006726 | eye pigment biosynthetic process | 2 | 1.053 | 0.040 |
| GO:0032446 | protein modification by small protein conjugation | 5 | 2.632 | 0.047 |
| GO:0001957 | intramembranous ossification | 2 | 1.053 | 0.050 |
| **GOTERM_CC_FAT** |  |  |  |  |
| GO:0009897 | external side of plasma membrane | 6 | 3.158 | 0.019 |
| GO:0044421 | extracellular region part | 15 | 7.895 | 0.052 |
| **GOTERM_MF_FAT** |  |  |  |  |
| GO:0008270 | zinc ion binding | 44 | 23.158 | 0.000 |
| GO:0046914 | transition metal ion binding | 48 | 25.263 | 0.000 |
| GO:0046872 | metal ion binding | 62 | 32.632 | 0.002 |
| GO:0043169 | cation binding | 62 | 32.632 | 0.002 |
| GO:0043167 | ion binding | 62 | 32.632 | 0.003 |
| GO:0005525 | GTP binding | 9 | 4.737 | 0.045 |
| GO:0017076 | purine nucleotide binding | 29 | 15.263 | 0.047 |
