# Supplementary material for: Identification of Biomarkers of Human Skin Ageing in Both Genders. Wnt Signalling - A Label of Skin Ageing?
Source: PLoS One. 2012 Nov 30;7(11):e50393. doi: 10.1371/journal.pone.0050393 (PMC3511529; doi:10.1371/journal.pone.0050393)
Supplement: Table S5 — Results of the analysis of statistically significant pathways at the <0.1 level in skin biopsies of females with age. Pathways were taken from the KEGG database (human pathways). Genes that were judged as non-detectable by the BG value criterion were excluded from analysis. (DOC) [file pone.0050393.s006.doc]

| **Females** |  | **# genes** | **Count %** | **P-value** |
| --- | --- | --- | --- | --- |
| KEGG_PATHWAY | Ribosome | 33 | 6.3 | 5.50E-25 |
| KEGG_PATHWAY | ECM-receptor interaction | 9 | 1.7 | 1.10E-02 |
| KEGG_PATHWAY | Focal adhesion | 14 | 2.7 | 2.90E-02 |
| KEGG_PATHWAY | Basal cell carcinoma | 6 | 1.1 | 4.80E-02 |
| KEGG_PATHWAY | Melanogenesis | 8 | 1.5 | 6.60E-02 |
| KEGG_PATHWAY | Colorectal cancer | 7 | 1.3 | 8.30E-02 |
| KEGG_PATHWAY | Hematopoietic cell lineage | 7 | 1.3 | 9.00E-02 |
| KEGG_PATHWAY | Wnt signaling pathway | 10 | 1.9 | 9.50E-02 |
| KEGG_PATHWAY | Cell adhesion molecules (CAMs) | 9 | 1.7 | 1.00E-01 |
| KEGG_PATHWAY | Leukocyte transendothelial migration | 8 | 1.5 | 1.30E-01 |
| KEGG_PATHWAY | Regulation of actin cytoskeleton | 12 | 2.3 | 1.50E-01 |
| KEGG_PATHWAY | Pathways in cancer | 16 | 3.1 | 2.10E-01 |
| KEGG_PATHWAY | Gap junction | 6 | 1.1 | 2.20E-01 |
| KEGG_PATHWAY | Alzheimer's disease | 9 | 1.7 | 2.40E-01 |
| KEGG_PATHWAY | Complement and coagulation cascades | 5 | 1 | 2.40E-01 |
| KEGG_PATHWAY | Retinol metabolism | 4 | 0.8 | 3.10E-01 |
| KEGG_PATHWAY | Antigen processing and presentation | 5 | 1 | 3.50E-01 |
| KEGG_PATHWAY | TGF-beta signaling pathway | 5 | 1 | 3.90E-01 |
| KEGG_PATHWAY | Lysosome | 6 | 1.1 | 4.20E-01 |
| KEGG_PATHWAY | Primary bile acid biosynthesis | 2 | 0.4 | 4.50E-01 |
| KEGG_PATHWAY | Long-term potentiation | 4 | 0.8 | 4.50E-01 |
| KEGG_PATHWAY | Bladder cancer | 3 | 0.6 | 4.50E-01 |
| KEGG_PATHWAY | Renal cell carcinoma | 4 | 0.8 | 4.70E-01 |
| KEGG_PATHWAY | ABC transporters | 3 | 0.6 | 4.80E-01 |
| KEGG_PATHWAY | Melanoma | 4 | 0.8 | 4.80E-01 |
| KEGG_PATHWAY | Systemic lupus erythematosus | 5 | 1 | 4.80E-01 |
| KEGG_PATHWAY | Endocytosis | 8 | 1.5 | 5.00E-01 |
| KEGG_PATHWAY | Notch signaling pathway | 3 | 0.6 | 5.10E-01 |
| KEGG_PATHWAY | B cell receptor signaling pathway | 4 | 0.8 | 5.10E-01 |
| KEGG_PATHWAY | Chronic myeloid leukemia | 4 | 0.8 | 5.10E-01 |
| KEGG_PATHWAY | Glycosaminoglycan degradation | 2 | 0.4 | 5.40E-01 |
| KEGG_PATHWAY | Insulin signaling pathway | 6 | 1.1 | 5.40E-01 |
| KEGG_PATHWAY | Endometrial cancer | 3 | 0.6 | 5.70E-01 |
| KEGG_PATHWAY | Nitrogen metabolism | 2 | 0.4 | 5.70E-01 |
| KEGG_PATHWAY | Vascular smooth muscle contraction | 5 | 1 | 5.80E-01 |
| KEGG_PATHWAY | Glycosylphosphatidylinositol(GPI)-anchor biosynthesis | 2 | 0.4 | 6.00E-01 |
| KEGG_PATHWAY | Pentose phosphate pathway | 2 | 0.4 | 6.00E-01 |
| KEGG_PATHWAY | Hedgehog signaling pathway | 3 | 0.6 | 6.10E-01 |
| KEGG_PATHWAY | ErbB signaling pathway | 4 | 0.8 | 6.20E-01 |
| KEGG_PATHWAY | Pathogenic Escherichia coli infection | 3 | 0.6 | 6.20E-01 |
| KEGG_PATHWAY | Prostate cancer | 4 | 0.8 | 6.30E-01 |
| KEGG_PATHWAY | Huntington's disease | 7 | 1.3 | 6.40E-01 |
| KEGG_PATHWAY | Dilated cardiomyopathy | 4 | 0.8 | 6.50E-01 |
| KEGG_PATHWAY | Asthma | 2 | 0.4 | 6.60E-01 |
| KEGG_PATHWAY | Glioma | 3 | 0.6 | 6.70E-01 |
| KEGG_PATHWAY | Chemokine signaling pathway | 7 | 1.3 | 6.80E-01 |
| KEGG_PATHWAY | Alanine. aspartate and glutamate metabolism | 2 | 0.4 | 6.80E-01 |
| KEGG_PATHWAY | Axon guidance | 5 | 1 | 6.90E-01 |
| KEGG_PATHWAY | GnRH signaling pathway | 4 | 0.8 | 7.00E-01 |
| KEGG_PATHWAY | Adipocytokine signaling pathway | 3 | 0.6 | 7.00E-01 |
| KEGG_PATHWAY | Long-term depression | 3 | 0.6 | 7.20E-01 |
| KEGG_PATHWAY | Basal transcription factors | 2 | 0.4 | 7.30E-01 |
| KEGG_PATHWAY | RIG-I-like receptor signaling pathway | 3 | 0.6 | 7.30E-01 |
| KEGG_PATHWAY | Viral myocarditis | 3 | 0.6 | 7.30E-01 |
| KEGG_PATHWAY | Allograft rejection | 2 | 0.4 | 7.40E-01 |
| KEGG_PATHWAY | Pancreatic cancer | 3 | 0.6 | 7.40E-01 |
| KEGG_PATHWAY | T cell receptor signaling pathway | 4 | 0.8 | 7.60E-01 |
| KEGG_PATHWAY | Graft-versus-host disease | 2 | 0.4 | 7.60E-01 |
| KEGG_PATHWAY | Arrhythmogenic right ventricular cardiomyopathy (ARVC) | 3 | 0.6 | 7.70E-01 |
| KEGG_PATHWAY | Calcium signaling pathway | 6 | 1.1 | 7.70E-01 |
| KEGG_PATHWAY | Fatty acid metabolism | 2 | 0.4 | 7.70E-01 |
| KEGG_PATHWAY | Aminoacyl-tRNA biosynthesis | 2 | 0.4 | 7.80E-01 |
| KEGG_PATHWAY | Type I diabetes mellitus | 2 | 0.4 | 7.90E-01 |
| KEGG_PATHWAY | N-Glycan biosynthesis | 2 | 0.4 | 8.20E-01 |
| KEGG_PATHWAY | Hypertrophic cardiomyopathy (HCM) | 3 | 0.6 | 8.20E-01 |
| KEGG_PATHWAY | Progesterone-mediated oocyte maturation | 3 | 0.6 | 8.20E-01 |
| KEGG_PATHWAY | Neurotrophin signaling pathway | 4 | 0.8 | 8.30E-01 |
| KEGG_PATHWAY | Intestinal immune network for IgA production | 2 | 0.4 | 8.40E-01 |
| KEGG_PATHWAY | Glutathione metabolism | 2 | 0.4 | 8.40E-01 |
| KEGG_PATHWAY | Autoimmune thyroid disease | 2 | 0.4 | 8.50E-01 |
| KEGG_PATHWAY | Parkinson's disease | 4 | 0.8 | 8.50E-01 |
| KEGG_PATHWAY | Oxidative phosphorylation | 4 | 0.8 | 8.60E-01 |
| KEGG_PATHWAY | Non-small cell lung cancer | 2 | 0.4 | 8.60E-01 |
| KEGG_PATHWAY | Cytosolic DNA-sensing pathway | 2 | 0.4 | 8.70E-01 |
| KEGG_PATHWAY | Tight junction | 4 | 0.8 | 8.70E-01 |
| KEGG_PATHWAY | Acute myeloid leukemia | 2 | 0.4 | 8.80E-01 |
| KEGG_PATHWAY | NOD-like receptor signaling pathway | 2 | 0.4 | 9.00E-01 |
| KEGG_PATHWAY | PPAR signaling pathway | 2 | 0.4 | 9.20E-01 |
| KEGG_PATHWAY | Jak-STAT signaling pathway | 4 | 0.8 | 9.20E-01 |
| KEGG_PATHWAY | VEGF signaling pathway | 2 | 0.4 | 9.40E-01 |
| KEGG_PATHWAY | Cell cycle | 3 | 0.6 | 9.50E-01 |
| KEGG_PATHWAY | Natural killer cell mediated cytotoxicity | 3 | 0.6 | 9.60E-01 |
| KEGG_PATHWAY | Apoptosis | 2 | 0.4 | 9.60E-01 |
| KEGG_PATHWAY | Ubiquitin mediated proteolysis | 3 | 0.6 | 9.60E-01 |
| KEGG_PATHWAY | MAPK signaling pathway | 6 | 1.1 | 9.70E-01 |
| KEGG_PATHWAY | Fc gamma R-mediated phagocytosis | 2 | 0.4 | 9.70E-01 |
| KEGG_PATHWAY | Toll-like receptor signaling pathway | 2 | 0.4 | 9.80E-01 |
| KEGG_PATHWAY | Oocyte meiosis | 2 | 0.4 | 9.80E-01 |
| KEGG_PATHWAY | Purine metabolism | 2 | 0.4 | 1.00E+00 |
| KEGG_PATHWAY | Neuroactive ligand-receptor interaction | 3 | 0.6 | 1.00E+00 |
| KEGG_PATHWAY | Cytokine-cytokine receptor interaction | 2 | 0.4 | 1.00E+00 |
| KEGG_PATHWAY | Epithelial cell signaling in Helicobacter pylori infection | 1 | 0.2 | 1.00E+00 |
| KEGG_PATHWAY | Linoleic acid metabolism | 1 | 0.2 | 1.00E+00 |
| KEGG_PATHWAY | Sulfur metabolism | 1 | 0.2 | 1.00E+00 |
| KEGG_PATHWAY | Amyotrophic lateral sclerosis (ALS) | 1 | 0.2 | 1.00E+00 |
| KEGG_PATHWAY | Porphyrin and chlorophyll metabolism | 1 | 0.2 | 1.00E+00 |
| KEGG_PATHWAY | Sphingolipid metabolism | 1 | 0.2 | 1.00E+00 |
| KEGG_PATHWAY | Glycerophospholipid metabolism | 1 | 0.2 | 1.00E+00 |
| KEGG_PATHWAY | Olfactory transduction | 1 | 0.2 | 1.00E+00 |
| KEGG_PATHWAY | Steroid hormone biosynthesis | 1 | 0.2 | 1.00E+00 |
| KEGG_PATHWAY | Fructose and mannose metabolism | 1 | 0.2 | 1.00E+00 |
| KEGG_PATHWAY | Fc epsilon RI signaling pathway | 1 | 0.2 | 1.00E+00 |
| KEGG_PATHWAY | Adherens junction | 1 | 0.2 | 1.00E+00 |
| KEGG_PATHWAY | Androgen and estrogen metabolism | 1 | 0.2 | 1.00E+00 |
| KEGG_PATHWAY | Valine. leucine and isoleucine degradation | 1 | 0.2 | 1.00E+00 |
| KEGG_PATHWAY | Arginine and proline metabolism | 1 | 0.2 | 1.00E+00 |
| KEGG_PATHWAY | Aldosterone-regulated sodium reabsorption | 1 | 0.2 | 1.00E+00 |
| KEGG_PATHWAY | Fatty acid elongation in mitochondria | 1 | 0.2 | 1.00E+00 |
| KEGG_PATHWAY | Selenoamino acid metabolism | 1 | 0.2 | 1.00E+00 |
| KEGG_PATHWAY | Lipoic acid metabolism | 1 | 0.2 | 1.00E+00 |
| KEGG_PATHWAY | Thyroid cancer | 1 | 0.2 | 1.00E+00 |
| KEGG_PATHWAY | RNA degradation | 1 | 0.2 | 1.00E+00 |
| KEGG_PATHWAY | Heparan sulfate biosynthesis | 1 | 0.2 | 1.00E+00 |
| KEGG_PATHWAY | Cardiac muscle contraction | 1 | 0.2 | 1.00E+00 |
| KEGG_PATHWAY | Spliceosome | 1 | 0.2 | 1.00E+00 |
| KEGG_PATHWAY | Proteasome | 1 | 0.2 | 1.00E+00 |
| KEGG_PATHWAY | Nucleotide excision repair | 1 | 0.2 | 1.00E+00 |
| KEGG_PATHWAY | Glycosphingolipid biosynthesis | 1 | 0.2 | 1.00E+00 |
| KEGG_PATHWAY | Chondroitin sulfate biosynthesis | 1 | 0.2 | 1.00E+00 |
| KEGG_PATHWAY | Vibrio cholerae infection | 1 | 0.2 | 1.00E+00 |
| KEGG_PATHWAY | Butanoate metabolism | 1 | 0.2 | 1.00E+00 |
| KEGG_PATHWAY | Taste transduction | 1 | 0.2 | 1.00E+00 |
| KEGG_PATHWAY | DNA replication | 1 | 0.2 | 1.00E+00 |
| KEGG_PATHWAY | Dorso-ventral axis formation | 1 | 0.2 | 1.00E+00 |
| KEGG_PATHWAY | Regulation of autophagy | 1 | 0.2 | 1.00E+00 |
| KEGG_PATHWAY | Phosphatidylinositol signaling system | 1 | 0.2 | 1.00E+00 |
| KEGG_PATHWAY | Glycine. serine and threonine metabolism | 1 | 0.2 | 1.00E+00 |
| KEGG_PATHWAY | Tyrosine metabolism | 1 | 0.2 | 1.00E+00 |
| KEGG_PATHWAY | One carbon pool by folate | 1 | 0.2 | 1.00E+00 |
| KEGG_PATHWAY | Prion diseases | 1 | 0.2 | 1.00E+00 |
| KEGG_PATHWAY | mTOR signaling pathway | 1 | 0.2 | 1.00E+00 |
| KEGG_PATHWAY | Small cell lung cancer | 1 | 0.2 | 1.00E+00 |
| KEGG_PATHWAY | Terpenoid backbone biosynthesis | 1 | 0.2 | 1.00E+00 |
| KEGG_PATHWAY | Arachidonic acid metabolism | 1 | 0.2 | 1.00E+00 |
| KEGG_PATHWAY | Histidine metabolism | 1 | 0.2 | 1.00E+00 |
| KEGG_PATHWAY | p53 signaling pathway | 1 | 0.2 | 1.00E+00 |
| KEGG_PATHWAY | SNARE interactions in vesicular transport | 1 | 0.2 | 1.00E+00 |
