# Supplementary material for: Identification of Biomarkers of Human Skin Ageing in Both Genders. Wnt Signalling - A Label of Skin Ageing?
Source: PLoS One. 2012 Nov 30;7(11):e50393. doi: 10.1371/journal.pone.0050393 (PMC3511529; doi:10.1371/journal.pone.0050393)
Supplement: Table S6 — Results of the analysis of statistically significant pathways at the <0.1 level in skin biopsies of males with age. Pathways were taken from the KEGG database (human pathways). Genes that were judged as non-detectable by the BG value criterion were excluded from analysis. (DOC) [file pone.0050393.s007.doc]

| **Males** |  | **# genes** | **Count %** | **P-value** |
| --- | --- | --- | --- | --- |
| KEGG_PATHWAY | Acute myeloid leukemia | 6 | 1.5 | 5.80E-03 |
| KEGG_PATHWAY | Melanogenesis | 7 | 1.8 | 1.40E-02 |
| KEGG_PATHWAY | Pathways in cancer | 12 | 3 | 6.30E-02 |
| KEGG_PATHWAY | Arginine and proline metabolism | 4 | 1 | 8.90E-02 |
| KEGG_PATHWAY | Gap junction | 5 | 1.2 | 1.00E-01 |
| KEGG_PATHWAY | Pathogenic Escherichia coli infection | 4 | 1 | 1.10E-01 |
| KEGG_PATHWAY | Cell adhesion molecules (CAMs) | 6 | 1.5 | 1.20E-01 |
| KEGG_PATHWAY | Alanine. aspartate and glutamate metabolism | 3 | 0.8 | 1.30E-01 |
| KEGG_PATHWAY | Basal transcription factors | 3 | 0.8 | 1.60E-01 |
| KEGG_PATHWAY | Wnt signaling pathway | 6 | 1.5 | 1.90E-01 |
| KEGG_PATHWAY | Vascular smooth muscle contraction | 5 | 1.2 | 1.90E-01 |
| KEGG_PATHWAY | Purine metabolism | 6 | 1.5 | 1.90E-01 |
| KEGG_PATHWAY | Colorectal cancer | 4 | 1 | 2.40E-01 |
| KEGG_PATHWAY | Apoptosis | 4 | 1 | 2.50E-01 |
| KEGG_PATHWAY | Intestinal immune network for IgA production | 3 | 0.8 | 2.60E-01 |
| KEGG_PATHWAY | Riboflavin metabolism | 2 | 0.5 | 2.80E-01 |
| KEGG_PATHWAY | Insulin signaling pathway | 5 | 1.2 | 2.90E-01 |
| KEGG_PATHWAY | Fc gamma R-mediated phagocytosis | 4 | 1 | 3.00E-01 |
| KEGG_PATHWAY | Pyrimidine metabolism | 4 | 1 | 3.00E-01 |
| KEGG_PATHWAY | Basal cell carcinoma | 3 | 0.8 | 3.00E-01 |
| KEGG_PATHWAY | Glycerophospholipid metabolism | 3 | 0.8 | 4.00E-01 |
| KEGG_PATHWAY | Long-term potentiation | 3 | 0.8 | 4.00E-01 |
| KEGG_PATHWAY | p53 signaling pathway | 3 | 0.8 | 4.00E-01 |
| KEGG_PATHWAY | Long-term depression | 3 | 0.8 | 4.00E-01 |
| KEGG_PATHWAY | Lysosome | 4 | 1 | 4.20E-01 |
| KEGG_PATHWAY | Thyroid cancer | 2 | 0.5 | 4.50E-01 |
| KEGG_PATHWAY | Glycine. serine and threonine metabolism | 2 | 0.5 | 4.70E-01 |
| KEGG_PATHWAY | Prion diseases | 2 | 0.5 | 5.10E-01 |
| KEGG_PATHWAY | Allograft rejection | 2 | 0.5 | 5.20E-01 |
| KEGG_PATHWAY | Ubiquitin mediated proteolysis | 4 | 1 | 5.20E-01 |
| KEGG_PATHWAY | Sphingolipid metabolism | 2 | 0.5 | 5.50E-01 |
| KEGG_PATHWAY | Fatty acid metabolism | 2 | 0.5 | 5.60E-01 |
| KEGG_PATHWAY | Tryptophan metabolism | 2 | 0.5 | 5.60E-01 |
| KEGG_PATHWAY | Type I diabetes mellitus | 2 | 0.5 | 5.70E-01 |
| KEGG_PATHWAY | GnRH signaling pathway | 3 | 0.8 | 5.90E-01 |
| KEGG_PATHWAY | Tyrosine metabolism | 2 | 0.5 | 5.90E-01 |
| KEGG_PATHWAY | ABC transporters | 2 | 0.5 | 5.90E-01 |
| KEGG_PATHWAY | Amino sugar and nucleotide sugar metabolism | 2 | 0.5 | 5.90E-01 |
| KEGG_PATHWAY | Toll-like receptor signaling pathway | 3 | 0.8 | 6.10E-01 |
| KEGG_PATHWAY | Cytokine-cytokine receptor interaction | 6 | 1.5 | 6.10E-01 |
| KEGG_PATHWAY | Autoimmune thyroid disease | 2 | 0.5 | 6.50E-01 |
| KEGG_PATHWAY | Endometrial cancer | 2 | 0.5 | 6.50E-01 |
| KEGG_PATHWAY | mTOR signaling pathway | 2 | 0.5 | 6.50E-01 |
| KEGG_PATHWAY | Huntington's disease | 4 | 1 | 7.10E-01 |
| KEGG_PATHWAY | Neurotrophin signaling pathway | 3 | 0.8 | 7.20E-01 |
| KEGG_PATHWAY | NOD-like receptor signaling pathway | 2 | 0.5 | 7.20E-01 |
| KEGG_PATHWAY | Axon guidance | 3 | 0.8 | 7.40E-01 |
| KEGG_PATHWAY | Adipocytokine signaling pathway | 2 | 0.5 | 7.50E-01 |
| KEGG_PATHWAY | PPAR signaling pathway | 2 | 0.5 | 7.60E-01 |
| KEGG_PATHWAY | Renal cell carcinoma | 2 | 0.5 | 7.60E-01 |
| KEGG_PATHWAY | Viral myocarditis | 2 | 0.5 | 7.70E-01 |
| KEGG_PATHWAY | Chronic myeloid leukemia | 2 | 0.5 | 7.80E-01 |
| KEGG_PATHWAY | VEGF signaling pathway | 2 | 0.5 | 7.80E-01 |
| KEGG_PATHWAY | B cell receptor signaling pathway | 2 | 0.5 | 7.80E-01 |
| KEGG_PATHWAY | MAPK signaling pathway | 5 | 1.2 | 7.90E-01 |
| KEGG_PATHWAY | Adherens junction | 2 | 0.5 | 7.90E-01 |
| KEGG_PATHWAY | Fc epsilon RI signaling pathway | 2 | 0.5 | 8.00E-01 |
| KEGG_PATHWAY | Antigen processing and presentation | 2 | 0.5 | 8.20E-01 |
| KEGG_PATHWAY | Small cell lung cancer | 2 | 0.5 | 8.20E-01 |
| KEGG_PATHWAY | Jak-STAT signaling pathway | 3 | 0.8 | 8.20E-01 |
| KEGG_PATHWAY | ErbB signaling pathway | 2 | 0.5 | 8.30E-01 |
| KEGG_PATHWAY | Prostate cancer | 2 | 0.5 | 8.40E-01 |
| KEGG_PATHWAY | Calcium signaling pathway | 3 | 0.8 | 8.70E-01 |
| KEGG_PATHWAY | Endocytosis | 3 | 0.8 | 8.90E-01 |
| KEGG_PATHWAY | T cell receptor signaling pathway | 2 | 0.5 | 8.90E-01 |
| KEGG_PATHWAY | Focal adhesion | 3 | 0.8 | 9.20E-01 |
| KEGG_PATHWAY | Regulation of actin cytoskeleton | 3 | 0.8 | 9.30E-01 |
| KEGG_PATHWAY | Neuroactive ligand-receptor interaction | 3 | 0.8 | 9.70E-01 |
| KEGG_PATHWAY | Olfactory transduction | 2 | 0.5 | 1.00E+00 |
| KEGG_PATHWAY | Tight junction | 1 | 0.2 | 1.00E+00 |
| KEGG_PATHWAY | Leukocyte transendothelial migration | 1 | 0.2 | 1.00E+00 |
| KEGG_PATHWAY | Glycosphingolipid biosynthesis | 1 | 0.2 | 1.00E+00 |
| KEGG_PATHWAY | Drug metabolism | 1 | 0.2 | 1.00E+00 |
| KEGG_PATHWAY | Epithelial cell signaling in Helicobacter pylori infection | 1 | 0.2 | 1.00E+00 |
| KEGG_PATHWAY | Inositol phosphate metabolism | 1 | 0.2 | 1.00E+00 |
| KEGG_PATHWAY | Glycolysis / Gluconeogenesis | 1 | 0.2 | 1.00E+00 |
| KEGG_PATHWAY | Pentose phosphate pathway | 1 | 0.2 | 1.00E+00 |
| KEGG_PATHWAY | Amyotrophic lateral sclerosis (ALS) | 1 | 0.2 | 1.00E+00 |
| KEGG_PATHWAY | Chemokine signaling pathway | 1 | 0.2 | 1.00E+00 |
| KEGG_PATHWAY | Phosphatidylinositol signaling system | 1 | 0.2 | 1.00E+00 |
| KEGG_PATHWAY | One carbon pool by folate | 1 | 0.2 | 1.00E+00 |
| KEGG_PATHWAY | Glutathione metabolism | 1 | 0.2 | 1.00E+00 |
| KEGG_PATHWAY | alpha-Linolenic acid metabolism | 1 | 0.2 | 1.00E+00 |
| KEGG_PATHWAY | TGF-beta signaling pathway | 1 | 0.2 | 1.00E+00 |
| KEGG_PATHWAY | RIG-I-like receptor signaling pathway | 1 | 0.2 | 1.00E+00 |
| KEGG_PATHWAY | ECM-receptor interaction | 1 | 0.2 | 1.00E+00 |
| KEGG_PATHWAY | Alzheimer's disease | 1 | 0.2 | 1.00E+00 |
| KEGG_PATHWAY | Spliceosome | 1 | 0.2 | 1.00E+00 |
| KEGG_PATHWAY | Melanoma | 1 | 0.2 | 1.00E+00 |
| KEGG_PATHWAY | Linoleic acid metabolism | 1 | 0.2 | 1.00E+00 |
| KEGG_PATHWAY | Arachidonic acid metabolism | 1 | 0.2 | 1.00E+00 |
| KEGG_PATHWAY | Mismatch repair | 1 | 0.2 | 1.00E+00 |
| KEGG_PATHWAY | SNARE interactions in vesicular transport | 1 | 0.2 | 1.00E+00 |
| KEGG_PATHWAY | RNA degradation | 1 | 0.2 | 1.00E+00 |
| KEGG_PATHWAY | Pancreatic cancer | 1 | 0.2 | 1.00E+00 |
| KEGG_PATHWAY | Systemic lupus erythematosus | 1 | 0.2 | 1.00E+00 |
| KEGG_PATHWAY | Retinol metabolism | 1 | 0.2 | 1.00E+00 |
| KEGG_PATHWAY | RNA polymerase | 1 | 0.2 | 1.00E+00 |
| KEGG_PATHWAY | Primary immunodeficiency | 1 | 0.2 | 1.00E+00 |
| KEGG_PATHWAY | Valine. leucine and isoleucine degradation | 1 | 0.2 | 1.00E+00 |
| KEGG_PATHWAY | DNA replication | 1 | 0.2 | 1.00E+00 |
| KEGG_PATHWAY | Steroid hormone biosynthesis | 1 | 0.2 | 1.00E+00 |
| KEGG_PATHWAY | Glycosphingolipid biosynthesis | 1 | 0.2 | 1.00E+00 |
| KEGG_PATHWAY | N-Glycan biosynthesis | 1 | 0.2 | 1.00E+00 |
| KEGG_PATHWAY | Oxidative phosphorylation | 1 | 0.2 | 1.00E+00 |
| KEGG_PATHWAY | Phenylalanine metabolism | 1 | 0.2 | 1.00E+00 |
| KEGG_PATHWAY | Hematopoietic cell lineage | 1 | 0.2 | 1.00E+00 |
| KEGG_PATHWAY | Cell cycle | 1 | 0.2 | 1.00E+00 |
| KEGG_PATHWAY | Asthma | 1 | 0.2 | 1.00E+00 |
| KEGG_PATHWAY | Graft-versus-host disease | 1 | 0.2 | 1.00E+00 |
| KEGG_PATHWAY | Drug metabolism | 1 | 0.2 | 1.00E+00 |
| KEGG_PATHWAY | Bladder cancer | 1 | 0.2 | 1.00E+00 |
| KEGG_PATHWAY | Chondroitin sulfate biosynthesis | 1 | 0.2 | 1.00E+00 |
| KEGG_PATHWAY | Glioma | 1 | 0.2 | 1.00E+00 |
| KEGG_PATHWAY | Aminoacyl-tRNA biosynthesis | 1 | 0.2 | 1.00E+00 |
| KEGG_PATHWAY | Homologous recombination | 1 | 0.2 | 1.00E+00 |
| KEGG_PATHWAY | Lysine degradation | 1 | 0.2 | 1.00E+00 |
| KEGG_PATHWAY | Histidine metabolism | 1 | 0.2 | 1.00E+00 |
| KEGG_PATHWAY | Complement and coagulation cascades | 1 | 0.2 | 1.00E+00 |
| KEGG_PATHWAY | Nucleotide excision repair | 1 | 0.2 | 1.00E+00 |
| KEGG_PATHWAY | Non-small cell lung cancer | 1 | 0.2 | 1.00E+00 |
| KEGG_PATHWAY | Fructose and mannose metabolism | 1 | 0.2 | 1.00E+00 |
| KEGG_PATHWAY | Ether lipid metabolism | 1 | 0.2 | 1.00E+00 |
| KEGG_PATHWAY | Cytosolic DNA-sensing pathway | 1 | 0.2 | 1.00E+00 |
| KEGG_PATHWAY | Base excision repair | 1 | 0.2 | 1.00E+00 |
| KEGG_PATHWAY | Natural killer cell mediated cytotoxicity | 1 | 0.2 | 1.00E+00 |
| KEGG_PATHWAY | Proteasome | 1 | 0.2 | 1.00E+00 |
| KEGG_PATHWAY | Glycosylphosphatidylinositol(GPI)-anchor biosynthesis | 1 | 0.2 | 1.00E+00 |
| KEGG_PATHWAY | Metabolism of xenobiotics by cytochrome P450 | 1 | 0.2 | 1.00E+00 |
| KEGG_PATHWAY | Sulfur metabolism | 1 | 0.2 | 1.00E+00 |
